# Single-cell CAS-seq reveals a class of short PIWI-interacting RNAs in human oocytes

**DOI:** 10.1038/s41467-019-11312-8

**Published:** 2019-07-29

**Authors:** Qiyuan Yang, Ronghong Li, Qifeng Lyu, Li Hou, Zhen Liu, Qiang Sun, Miao Liu, Huijuan Shi, Beiying Xu, Mingru Yin, Zhiguang Yan, Ying Huang, Mofang Liu, Yiping Li, Ligang Wu

**Affiliations:** 1 State Key Laboratory of Molecular Biology, CAS Center for Excellence in Molecular Cell Science, Institute of Biochemistry and Cell Biology, Chinese Academy of Sciences, University of Chinese Academy of Sciences, Shanghai, 200031 China; 2 Shanghai Key Laboratory of Molecular Andrology, CAS Center for Excellence in Molecular Cell Science, Institute of Biochemistry and Cell Biology, Chinese Academy of Sciences, University of Chinese Academy of Sciences, Shanghai, 200031 China; 3 Department of Assisted Reproduction, Shanghai Ninth People’s Hospital, Shanghai Jiaotong University School of Medicine, Shanghai, 200011 China; 4 Institute of Neuroscience, CAS Key Laboratory of Primate Neurobiology, State Key Laboratory of Neuroscience, CAS Center for Excellence in Brain Science and Intelligence Technology, Shanghai Institutes for Biological Sciences, Chinese Academy of Sciences, Shanghai, 200031 China; 5 China National Population and Family Planning Key Laboratory of Contraceptive Drugs and Devices, Shanghai Institute of Planned Parenthood Research (SIPPR), Shanghai, 200032 China; 6 Shanghai Key Laboratory of Reproductive Medicine, Shanghai Jiao Tong University, Shanghai, 200025 China

**Keywords:** RNA sequencing, Small RNAs, Embryology, Gene expression

## Abstract

Small RNAs have important functions. However, small RNAs in primate oocytes remain unexplored. Herein, we develop CAS-seq, a single-cell small RNA sequencing method, and profile the small RNAs in human oocytes and embryos. We discover a class of ~20-nt small RNAs that are predominantly expressed in human and monkey oocytes, but not in mouse oocytes. They are specifically associated with HIWI3 (PIWIL3), whereas significantly shorter than the commonly known PIWI-interacting RNAs (piRNAs), designated as oocyte short piRNAs (os-piRNAs). Notably, the os-piRNAs in human oocytes lack 2’-O-methylation at the 3’ end, a hallmark of the classic piRNAs. In addition, the os-piRNAs have a strong 1U/10 A bias and are enriched on the antisense strands of recently evolved transposable elements (TEs), indicating the potential function of silencing TEs by cleavage. Therefore, our study has identified an oocyte-specific piRNA family with distinct features and provides valuable resources for studying small RNAs in primate oocytes.

## Abstract (web summary)

PIWI-interacting RNAs (piRNAs) are ~25–33 nt small RNAs expressed in animal germ cells. Here, the authors develop a single-cell small RNA sequencing method and report that a class of ~20-nt piRNAs lacking 3′ end 2′-O-methylation are associated with PIWIL3 protein and predominantly expressed in human and monkey oocytes.

## Introduction

MicroRNAs (miRNAs), endogenous small interfering RNAs (endo-siRNAs), and PIWI-interacting RNAs (piRNAs) are the major types of small RNAs present in mammalian oocytes and early embryos^1^. miRNAs and endo-siRNAs are ~22 nt long and are associated with Argonaute (AGO) proteins^2,3^. While miRNAs are processed sequentially from hairpin structures embedded in long transcripts by Drosha and Dicer^4–6^, endo-siRNAs are usually derived from endogenous, long RNA duplexes after being cleaved by Dicer^7–9^. The knockout of Dicer or Ago2 in mouse oocytes leads to severe chromatin segregation defects during meiosis and causes female sterility^10,11^. piRNAs are germ cell-specific small RNAs that form clusters at hundreds of genome locations and are functional in protecting the animal’s germline by silencing transposons and regulating gene expression^7,12–14^.

The biogenesis of piRNAs markedly differs from that of miRNAs and endo-siRNAs. piRNAs are first processed from primary transcripts by the nuclease Zucchini^15,16^. The piRNA intermediates bind to the PIWI proteins and are trimmed to lengths of 25–33 nt from the 3′ end^17^; Hua enhancer 1 (Hen1) further 2′-O-methylates them at their 3′ termini^18,19^. PIWI proteins, in complex with primary piRNAs, cleave target RNAs between positions 10 and 11 of the complementary sequence. Such cleavage not only destroys the transposon RNAs but also produces the sense-strand piRNA intermediates that bind to the PIWI proteins. These intermediates are further trimmed by a 3′ exonuclease to generate secondary piRNAs in a mechanism known as the “ping-pong” model for piRNA biogenesis^12–14,19–21^. While the PIWI proteins have a strong bias for uracil (U) at the 5′ ends of the piRNAs they bind, the secondary piRNAs usually have additional features, including an A residue at position 10 (10A) and a 10-nt 5′- end overlap with the primary piRNAs. Due to their germline expression, disruption of PIWI in multiple species has varying implication for reproduction. In Drosophila, the disruption of PIWI causes sterility in both females and males^22^, and the disruption of AGO3 causes sterility in females and semi-sterility in males^23^. In zebrafish, the disruption of the piRNA pathway causes both female and male sterility^24^. Most mammalian genomes encode four PIWI proteins (PIWIL1–4), whereas mice and rats lack PIWIL3, and only encode PIWIL1, PIWIL2, and PIWIL4. Knockout of any of the three *Piwi* genes in mice causes sterility exclusively in males^25^. These species-dependent differences in the impact of PIWI loss raise the question of whether piRNAs have important functions in mammalian female germ cells.

Many studies have demonstrated that small RNAs play critical roles in germ cell development in model animals^11,26,27^; however, the profiles of small RNAs in primate oogenesis and in early embryos remain unclear due to the technical obstacles in sequencing small RNAs with an extremely limited amount of input RNA. Herein, we describe a highly sensitive single-cell small RNA-sequencing (RNA-seq) method suitable for detecting low-copy small RNAs and utilize this method to profile small RNAs in human oocytes and early embryos.

## Results

### CAS-seq development for single-cell small RNA-seq

**Fig. 1:**
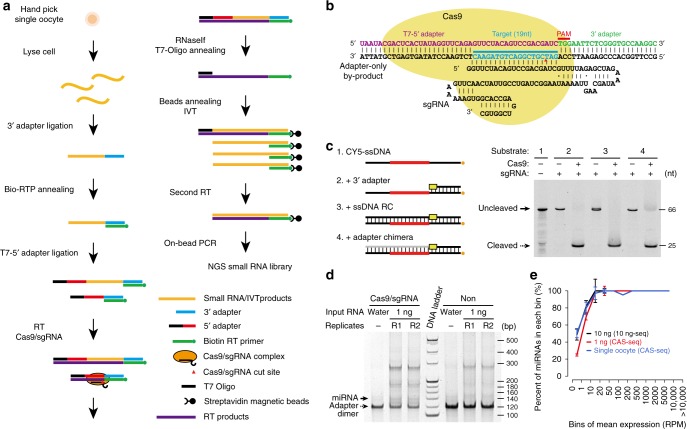
Cas9-assisted sequencing of small RNAs. **a** An overview of the CAS-seq (Cas9-assisted small RNA-sequencing) method. RNA (yellow) from an individual oocyte was ligated sequentially with a 3′ adapter (blue) and a T7-5′ adapter (black and red). The ligation products were reverse-transcribed using a biotin-labelled reverse transcription (RT) primer (green), followed by Cas9/single guide RNA (sgRNA) cleavage to eliminate adapter heterodimers. The RT products were coupled to streptavidin beads for in vitro transcription (IVT) linear amplification. After a second round of reverse transcription, the beads were used directly for PCR amplification, and the library was size selected by polyacrylamide gel electrophoresis for next-generation sequencing. IVT, in vitro transcription. **b** Diagram of the Cas9-sgRNA designed to specifically recognize and cleave the RT products of 5′ and 3′ adapter heterodimers. T7-5′ adapter (RNA oligo, purple); 3′ adapter (DNA oligo, green); DNA sequence targeted by sgRNA (DNA oligo, blue); PAM sequence (TGG at the 5′ end of the 3′ adapter). **c** Denatured polyacrylamide gel electrophoresis (PAGE) analysis of Cas9-sgRNA cleavage products. The substrates of Cas9/sgRNA are shown on the left. DNA oligo (black), PAM sequence (yellow box), sgRNA target region (red), RNA oligo (gray), and CY5 fluorescence (orange). **d** Comparison of small RNA library construction results with or without Cas9/sgRNA treatment. One nanogram of human embryonic kidney 293 (HEK293) total RNA was used as the input, and the PCR amplification products were analyzed by 6% PAGE. **e** Sensitivity comparison of CAS-seq and other methods to detect microRNAs (miRNAs) in mouse oocytes. Total RNA (1 ng) from mouse oocytes (red) and a single mouse oocyte (blue) were sequenced by CAS-seq, and 10 ng of total RNA from mouse oocytes (black) was sequenced using our previously reported 10 ng sequencing protocol. The mean and standard deviation of the percentage of miRNAs detected by each method are shown. Source data of **c**–**e** are provided in the Source Data file

The efficient ligation of adapters to scarce small RNAs requires a high concentration of 5′ and 3′ adapters. This requirement produces a high level of adapter heterodimer by-products, which hinder the subsequent amplification of the small RNA complementary DNA (cDNA) libraries^28^. The single guide RNA (sgRNA)-guided Cas9 nuclease (spCas9) is capable of cleaving target double-stranded DNA (dsDNA) bearing a protospacer adjacent motif (PAM) sequence both in vitro and in vivo^29,30^. The 5′ and 3′ adapter heterodimer is RNA–DNA chimera (Fig. 1a, b) and is not a canonical substrate able to be cleaved by spCas9. We found that in the presence of the cDNA strand generated by reverse transcription (RT), spCas9 can cleave the RNA–DNA/cDNA chimeras bearing a PAM sequence (TGG) in the 3′ adapter sequence with comparable efficiency to its dsDNA substrates (Fig. 1b, c, Supplementary Fig. 1a). Treatment with Cas9-sgRNA significantly reduced the level of adapter heterodimers and enhanced the amplification of the cDNA, allowing the miRNA products (approximately 140 bp) to easily be detected by electrophoresis on a polyacrylamide gel (Fig. 1d). To suppress bias during exponential amplification by PCR, we introduced an in vitro transcription (IVT) linear amplification step that efficiently reduced the PCR amplification by ten cycles^31^ (Supplementary Fig. 1b). To avoid extracting total RNAs from a single cell, which is technically challenging and usually causes a significant loss of RNA content, we used heat to lyse the cell and to release the small RNAs from RNA–protein complexes before ligation with a 3′ adapter. We also optimized this procedure by conducting multiple enzymatic reactions on beads. With all of these efforts, we developed CAS-seq (Cas9-assisted small RNA-sequencing) and were able to reduce the input of total RNA to 1 ng or less. The sequencing results faithfully recapitulated (*R* > 0.87) the expression of miRNAs in human embryonic kidney 293 (HEK293) cells as well as mouse oocytes without obvious off-target events (Supplementary Fig. 1c–e, Supplementary Data 1). The accuracy of the sequencing results was further validated by quantitative RT-PCR (qRT-PCR) (Supplementary Fig. 1f). We further sequenced small RNAs in mouse testes, which contain highly diverse small RNA populations such as piRNAs (Supplementary Fig. 2a). Although the coverage of piRNAs in piRNA clusters detected by CAS-seq using 1 ng of total RNA was less than that by TruSeq using 200 ng of total RNA (Supplementary Fig. 2b, c), most of the piRNAs and piRNA clusters could be detected by CAS-seq (Supplementary Fig. 2d, e) and the relative expression level of piRNAs from each piRNA cluster was faithfully recapitulated (Supplementary Fig. 2f). An additional treatment with 5′ deadenylase and exonuclease (CAS-seq-2E) to eliminate remnant 3′ adapters could further reduce the minimum RNA input requirement. The sequencing results of 1 ng testes RNA by CAS-seq-2E were similar to those of CAS-seq (Supplementary Fig. 2g), indicating that the method is reliable. This result allowed us to profile even more challenging samples such as small RNAs in an immunoprecipitation (IP) experiment with only a few cells.

### Sequencing small RNAs in human oocytes by CAS-seq

**Fig. 2:**
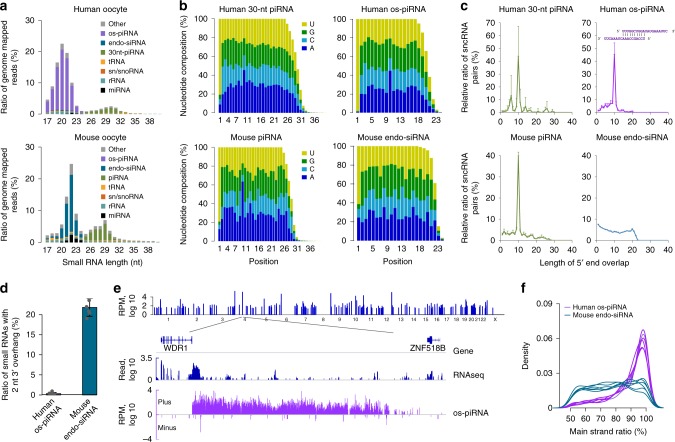
Characteristics of novel 20-nucleotide (nt) small RNAs (oocyte short-PIWI-interacting RNAs (os-piRNAs)) in human oocytes. **a** Composition of small RNA categories according to their length distributions in both human and mouse oocytes. The length of os-piRNAs in human oocytes was ~2 nt shorter than that of mouse endo-small interfering RNAs (endo-siRNAs). **b** The relative nucleotide composition of piRNAs, endo-siRNAs, and os-piRNAs in **a**. Both os-piRNAs and the 30-nt piRNAs in human oocytes, as well as the piRNAs in mouse oocytes, have a preference of 1U and 10A in nucleotide compositions. **c** Length distribution of 5′ overlaps between small RNA pairs mapped to the opposite genomic strands of the same locus. The spike at 10 nt indicates the signature of a 10-nt 5′-end overlap between the partially complementary small RNA pairs. An example of an os-piRNA pair with a 10-nt 5′-end overlap signature is shown. **d** The proportion of complementary reads with a 2-nt 3′ overhang in human os-piRNAs was significantly lower than that in mouse endo-siRNAs. The average result in six human/mouse oocyte samples is shown in **a**–**d**, and the error bars in **c**, **d** represent the standard deviation. **e** Distribution of os-piRNA clusters on human chromosomes (top) and an instance of an os-piRNA cluster (bottom). The positive and negative numbers of the vertical axis represent the expression on the plus and minus strands, respectively. **f** Human os-piRNAs were more prone to be derived from single-stranded precursors compared to mouse endo-siRNAs. Strand bias in six replicate samples is shown. Source data of **a**–**f** are provided in the Source Data file

We used CAS-seq to profile the small RNAs in single oocytes of *Mus musculus* (Supplementary Data 2). The sequencing results of the biological replicates of single mouse oocytes were highly reproducible (*R* > 0.94) (Supplementary Fig. 3a), and the detection sensitivity was similar to the results generated using 10 ng of total oocyte RNA (Fig. 1e, Supplementary Data 3). The major types of small RNAs detected in the mouse oocytes were endo-siRNAs (~22 nt), piRNAs (~29 nt), and miRNAs (~22 nt) (Fig. 2a); this outcome is consistent with the results of previous deep-sequencing studies using bulk purified total RNA^8,28,32^, which further validates the reliability of our method. Then, we used CAS-seq to profile the small RNAs in single oocytes of *Homo sapiens*. The length distribution of the small RNAs in human germinal vesicle (GV) and metaphase II (MII) stage oocytes showed a bi-modal pattern similar to that of mouse oocytes (Fig. 2a, Supplementary Fig. 3c). No significant changes in the composition of these small RNAs were observed in in vitro growing human oocytes (Supplementary Fig. 3d). The peak centered at 30 nt corresponded to the piRNAs (30-nt piRNAs) and had features similar to the typical piRNA previously identified in human testes and ovaries^33,34^, including a high sequence diversity, clustering on the genome, the sequence preference of 1U/10A, and 10-nt 5′-end overlap between the complementary piRNA pairs (Fig. 2b, c, Supplementary Data 4–5). The miRNAs accounted for <2% of the total reads mapped to the human genome and showed a significant correlation with those expressed in mouse oocytes (Supplementary Fig. 3e). Surprisingly, human oocytes contained a distinct category of previously uncharacterized small RNAs that were approximately 20 nt long (2 nt shorter than miRNAs and endo-siRNAs) and that accounted for >70% of the total small RNAs expressed in oocytes (Fig. 2a). These abundant 20-nt small RNAs were not present in human cumulus cells (Supplementary Fig. 3f), nor were reported previously in other human tissues. They were also readily detected using the total RNA extracted from multiple human oocytes (Supplementary Fig. 3c, g). This finding indicates that these RNAs are not contamination from surrounding cells and are not the RNA degradation products generated after cell lysis during library construction from the single oocyte. The sequences of these small RNAs are highly diverse, and hundreds of thousands of different sequences can be readily detected in a single human oocyte (Supplementary Data 6). RNA secondary structure predictions showed that they cannot form stable hairpin structures with their flanking sequences, indicating that they were not products of Drosha/DGCR8 cleavage and were unlikely related to miRNAs. They also lacked complementary strands with a 2-nt 3′ overhang (Fig. 2d), a typical signature of small RNA duplexes processed by Dicer, suggesting that they also do not belong to the endo-siRNA class that is predominantly present in mouse oocytes. Intriguingly, they tended to be located in specific loci and formed more than 200 clusters on the genome, ranging from a couple kilobases (kb) to hundreds of kb in length (Fig. 2e, Supplementary Fig. 3g, h, Supplementary Data 7). The distribution of these 20-nt small RNAs in these clusters had strong strand asymmetry (Fig. 2f), supporting their single-strand origin. These 20-nt small RNAs had a preponderant bias for uracil at the first position (1U) and a strong bias for adenine at the tenth position (10A) (Fig. 2b), as well as a signature of a 10-nt 5′-end overlap between these small RNA pairs mapped to the opposite genomic strands (Fig. 2c); these characteristics are reminiscent of the sequence features of piRNAs in mammalian testes^35–38^.

### 20-nt Oocyte small RNAs associate with HIWI3 in human oocytes

**Fig. 3:**
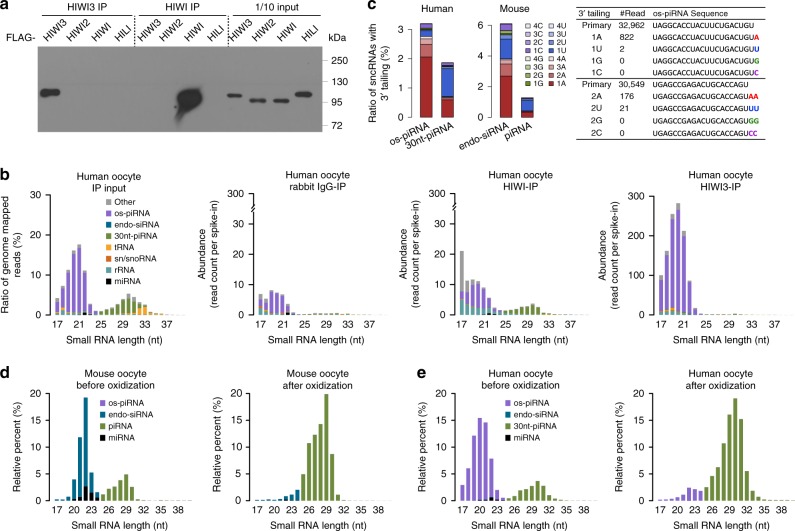
The interacting protein and 3′ modification of oocyte short-PIWI-interacting RNAs (os-piRNAs). **a** Specificity of anti-HIWI and anti-HIWI3 antibodies. FLAG-tagged HIWI3, HIWI2, HIWI, and HILI were ectopically expressed in HEK293 cells. The cell lysates were immunoprecipitated with HIWI3- or HIWI-specific antibodies and immunoblotted with an anti-FLAG antibody. **b** os-piRNAs were enriched by HIWI3 immunoprecipitation (IP) in human oocytes. The abundance and length distribution of small RNAs in the input and immunoprecipitated (IP) samples of oocytes are shown. Three or four human oocytes were used for each IP reaction. Rabbit nonspecific immunoglobulin G (IgG) antibody served as a negative control. The abundance of small RNAs was normalized to an exogenous spike-in. **c** The ratio of small RNAs tailed with mono- and oligo-nucleotides in human and mouse oocytes is shown. The instances of 3′ adenylation in os-piRNAs are shown. **d**, **e** Composition of small RNAs in the mouse and human oocytes before and after NaIO_4_ oxidization. The average result of three biological replicates is shown. Source data of **a**–**e** are provided in the Source Data file

The human genome encodes four PIWI proteins as follows: HIWI (PIWIL1), HILI (PIWIL2), HIWI3 (PIWIL3), and HIWI2 (PIWIL4), and four AGO proteins (AGO1–4). We generated PIWI antibodies against short peptide sequences in order to perform IP experiments to identify protein interactions with these 20-nt small RNAs. Previous transcriptomic studies have revealed that HIWI2 is rarely expressed in human oocytes (Supplementary Fig. 4a)^39,40^; therefore, we did not make the extra effort to generate a HIWI2 antibody. The specificity of the HIWI, HIWI3, and HILI antibodies were verified by IP assays of the FLAG-tagged human PIWI proteins ectopically expressed in HEK293 cells (Fig. 3a and Supplementary Fig. 4b). In addition, endogenous HIWI (861 aa, ~95 kDa) and HILI (973 aa, ~107 kDa) proteins were detected in the lysate of human adult testes by western blot using their corresponding antibodies, whereas no HIWI3 protein expression was detected (Supplementary Fig. 4c). This result is consistent with PIWI messenger RNA (mRNA) expression in human testes (Supplementary Fig. 4d), further confirming the specificity of these antibodies. We performed IP experiments of endogenous PIWI proteins using human adult testis lysates and the results showed that HIWI bound to ~30-nt piRNAs and HILI bound to ~26-nt piRNAs (Supplementary Fig. 4e, f). Few small RNAs could be detected by HIWI3 IP experiments in the human testes, which was likely due to the lack of HIWI3 expression. These observations are consistent with the previous studies of PIWI protein expression and the length of the piRNAs they bind to in human and mouse testes^34,41^, further validating the selectivity of the antibodies we generated. We also conducted an IP assay for human oocytes with AGO- and PIWI-specific antibodies and sequenced the small RNAs they bound to. In human oocytes, miRNAs were readily precipitated with AGO1 and AGO2, whereas the small RNAs bound to AGO3 and AGO4 were indistinguishable from those bound to the negative control immunoglobulin G (IgG) antibody (Fig. 3b, Supplementary Fig. 4g), which was likely due to the low expression level of AGO3 and AGO4 in human oocytes (Supplementary Fig. 4a)^39^. To quantify the enrichment of small RNAs, the relative abundance of the 20-nt small RNAs vs. the 30-nt piRNAs that immunoprecipitated with individual PIWI-specific antibodies was compared to those immunoprecipitated with the negative control IgG antibody or oocytes lysate (IP input). Intriguingly, the relative ratio of 20-nt small RNAs to 30-nt piRNAs specifically bound to HIWI3 showed an enrichment of 5.4-fold over IgG-IP and 28.3-fold over the IP input (Fig. 3b, Supplementary Fig. 4h). IP using an anti-HILI antibody in human oocytes did not generate sufficient sequence reads for a reliable analysis, probably due to the relatively low expression of HILI and no obvious expression of the 26-nt piRNAs in human oocytes. HIWI is also expressed in human oocytes, albeit at a relatively lower level than that of HIWI3 as reported previously (Supplementary Fig. 4a). The relative ratio of 30-nt piRNAs to 20-nt small RNAs specifically bound to HIWI showed an enrichment of 7.6-fold over IgG-IP and 1.5-fold over the IP input (Fig. 3b, Supplementary Fig. 4h), indicating that HIWI preferentially binds to 30-nt piRNAs, although we could not rule out the possibility that a small portion of the 20-nt small RNAs may also bind to HIWI.

The length of these HIWI3-associated 20-nt small RNAs was significantly shorter than that of previously reported piRNAs in the human ovaries and testes that bound to HIWI (~30 nt), HILI (~26 nt), and HIWI2 (~28 nt)^34^ or that of the piRNAs identified in other mammals. We named these ~20-nt HIWI3-interacting small RNAs with a 1U/10A signature and an abundant expression in human oocytes as: human oocyte short-PIWI-interacting small RNAs (os-piRNAs). A comparable type of 20-nt os-piRNA was absent in mouse oocytes, which may be due to a lack of a HIWI3 paralog in the mouse genome (Fig. 2a, Supplementary Fig. 3b). Consistent with the presence of a 10-nt 5′-end overlap signature in the 20-nt piRNAs (Supplementary Fig. 4i), the DEDH site in the PIWI domain of HIWI3 is conserved and remains intact (Supplementary Fig. 5a), suggesting that the slicer activity of HIWI3 might be required for the biogenesis and function of os-piRNAs. Although the 30-nt piRNAs were abundant in human oocytes, they did not bind to HIWI3 (Fig. 3b), suggesting that os-piRNAs are not likely to be derived from 30-nt piRNAs by trimming the 3′ end. More than 80% of the os-piRNA clusters had corresponding long transcripts that was previously detected by RNA-seq data from human oocytes^39,40^. Although the abundance of individual os-piRNAs varied in replicate samples, the total os-piRNAs generated from each cluster were consistent (Supplementary Fig. 5b). We found no significant correlation between the abundance of os-piRNAs and their precursors (Supplementary Fig. 5c), suggesting that the os-piRNAs are not likely to be the degradation production from long transcripts. Previous studies of piRNA processing in mouse testes showed that the distance from the 5′ ends of upstream piRNAs to the 5′ ends of downstream piRNAs (5′-to-5′ distances) was ~35 nt^42–45^, indicating the presence of phasing and 3′ trimming during the processing of piRNAs^46–48^. Intriguingly, we found that the 5′-to-5′ distances for the os-piRNA was ~20 nt and that the distance for the 30-nt piRNA was ~30 nt (Supplementary Fig. 5d), suggesting that the processing of human oocyte piRNAs also has a phasing pattern but without significant 3′ trimming, which is similar to the pattern of piRNA processing in *Drosophila* ovaries^42,43^. However, we could not fully rule out the possibility that the sensitivity of our current single-cell sequencing method may not be sufficient to detect low levels of trimming signatures. Notably, the nucleotide references and the relative ratios of the 3′ tailing were significantly different in os-piRNAs and the 30-nt piRNAs (Fig. 3c). The ratio of 3′ adenylation in the os-piRNAs was much higher than that in the 30-nt piRNAs. In contrast, uracil (U) was found most often to be added to the 3′ end of 30-nt piRNAs, indicating that os-piRNAs and 30-nt piRNAs are processed differently in human oocytes. With all of these observations, we speculate that os-piRNAs are processed from long transcript precursors rather than being the degradation or trimming products of 30-nt piRNAs, although we cannot completely exclude other possibilities.

### os-piRNAs lack 2′-O-methylation at their 3′ terminus

The classical piRNAs are 25–33 nt in length and contain 2′-O-methylation at their 3′ terminus, a hallmark feature of piRNA, which is added by the small RNA methyltransferase Hen1^49–51^ to protect the piRNAs from 3′ trimming and degradation^52,53^. Treatment of small RNAs with NaIO_4_ before cDNA library construction can oxidize the free 2′-OH and 3′-OH groups on their last nucleotides, thus enriching the small RNAs with 3′ end 2′-O-methylation^54^. We optimized the oxidation experiment conditions for a small amount of total RNA, which allowed us to analyze the 3′ methylation status of small RNAs in a few oocytes (Fig. 3d, e). As expected, the endo-siRNAs and miRNAs in the mouse oocytes (Fig. 3d) and testes (Supplementary Fig. 5e) were diminished after oxidation, whereas the classic piRNAs remained. This result is consistent with previous studies and validated the reliability of our assay. Surprisingly, the os-piRNAs in human oocytes almost completely disappeared after oxidation, indicating that os-piRNAs lack 2′-O-methylation at their 3′ end (Fig. 3e). In contrast, the long piRNAs (>24 nt) in human oocytes and testes remained intact. These observations suggest that the biogenesis pathway of os-piRNAs and long piRNAs might be different. The shorter length of os-piRNAs might restrict the accessibility of RNA methyltransferases when they bind to PIWI protein. Alternatively, HIWI3 or its associated proteins might lack the ability to recruit RNA methyltransferase Hen1 to catalyze the 2′-O-methylation at the 3′ end of os-piRNAs.

### os-piRNAs target transposable elements

**Fig. 4:**
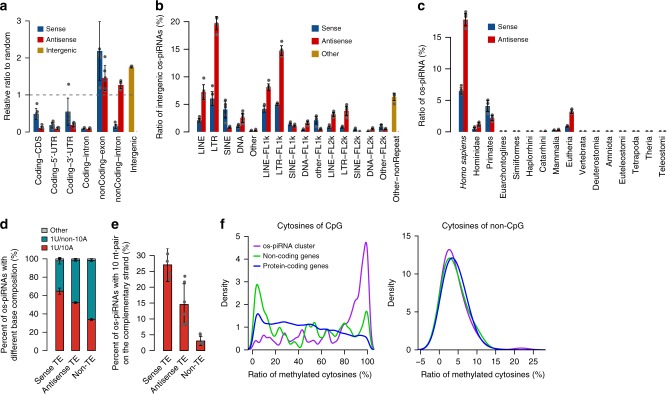
Potential function of oocyte short-PIWI-interacting RNAs (os-piRNAs). **a** Enrichment of human os-piRNAs in different gene elements compared to the random genomic distribution. **b** Proportion of intergenic os-piRNAs on the sense and antisense strand of different transposable elements (TEs) and their flanking regions. FL1k and FL2k represent the 0–1- and 1–2-kb flanking regions, respectively. **c** The proportion of os-piRNAs on the sense and antisense strand of transposons is classified according to their evolutionary origin. **d** The ratio of reads with both 1U and 10A in os-piRNAs derived from sense TEs is greater than those from antisense TEs or non-TE regions. **e** os-piRNAs derived from sense TEs contained more 10 nt pairs on the complementary strand. Average results in the six replicate samples of human oocytes are shown for **a**–**e**, and the error bars represent the standard deviation. **f** The density plot of the CpG and non-CpG methylation ratios of os-piRNA clusters, protein-coding genes, and non-coding genes. Source data of **a**–**f** are provided in the Source Data file

Compared to a random distribution, the os-piRNAs were enriched in non-coding genes and intergenic regions, less enriched in untranslated regions (UTRs) and coding sequences (CDSs), and were rare in the intronic regions of protein-coding genes (Fig. 4a). Notably, more than 85% of the os-piRNAs were derived from unannotated transcripts located in intergenic regions (Supplementary Fig. 6a). The intergenic os-piRNAs were enriched on the antisense strand of transposable elements (TEs) and at their flanking regions (Fig. 4b, Supplementary Fig. 6b, c). By contrast, while most of the endo-siRNAs and piRNAs in the mouse oocytes were derived from TEs, they were rarely derived from their flanking region and showed no significant TE-antisense enrichment (Supplementary Fig. 6d), illustrating that the mechanisms for small RNA processing and TE silencing are species-specific. Among the transposons related to os-piRNAs, most were newly evolved in *Homo sapiens* during evolution (Fig. 4c), suggesting a potential function of os-piRNAs in targeting the transcripts of active TEs. The os-piRNAs derived from the sense strand of TEs have a significantly higher level of 10A bias and 10-nt 5′-end overlap signature than those derived from the antisense strand of TEs and non-TEs, indicating effective cleavage of TE transcripts guided by TE-antisense os-piRNAs in the oocytes (Fig. 4d, e). This phenomenon is reminiscent of the function of testes piRNAs in silencing the TE transcripts in male germ cells by the slicer activity of PIWI family proteins in mice^55,56^. In addition, more than 99% of the uniquely mapped os-piRNAs with the ping-pong signatures were derived from non-coding regions in the human genome, and only ~0.8% of them were derived from protein-coding transcripts. Most of the os-piRNAs that map to the protein-coding transcripts are sense strands, suggesting that os-piRNAs are not inclined to regulate mRNA expression through ping-pong processing. However, we cannot rule out the possibility that the mRNAs bearing TE-related sequences in the UTR might be silenced by complementary TE-derived piRNAs. We further analyzed the previously published DNA methylation data of human oocytes^57^ and found that the average DNA CpG methylation level of os-piRNA clusters were significantly higher than that of the protein-coding and non-coding genes (Fig. 4f), which may serve as a genomic marker for the transcription or processing of piRNA clusters. Intriguingly, some of the highly expressed os-piRNA clusters were found to partially overlap with maternally imprinted genes such as KCNK9, indicating that os-piRNAs may also play a role in maintaining the methylation pattern of maternal genomic DNA.

**Fig. 5:**
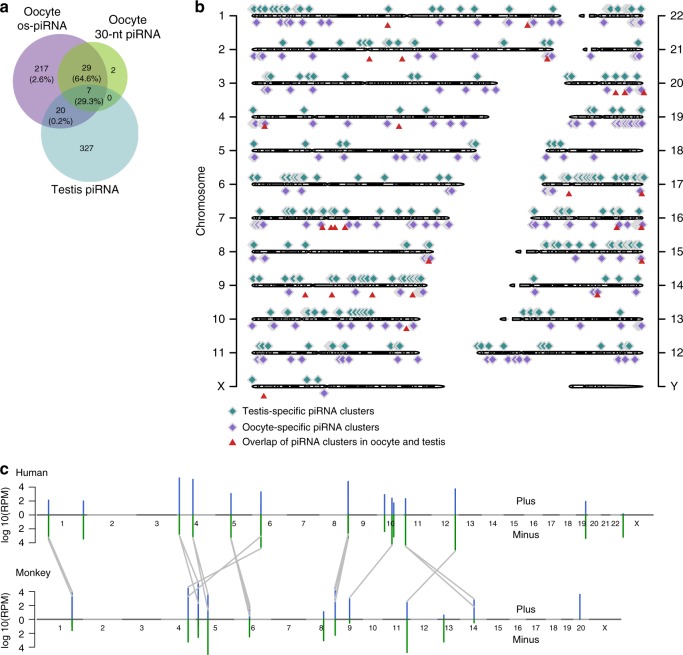
Conservation of oocyte short-PIWI-interacting RNAs (os-piRNAs) in human and monkey. **a** The amount of overlap among the os-piRNA clusters in human oocytes, the 30-nt piRNA clusters in human oocytes, and the piRNA clusters in human testes. The numbers in the brackets represent the ratios of the os-piRNAs expressed in these overlapped clusters in human oocytes. **b** Locations of oocyte- and testis-piRNA clusters on human chromosomes. The os-piRNA and the 30-nt piRNA clusters in human oocytes were combined as piRNA clusters in human oocytes. **c** The orthologous relationship between human and monkey os-piRNA clusters. The chromosomes of each species are represented vertically from chromosome 1 to the sex chromosomes. Gray lines indicate orthologous cluster pairs. Clusters with an average reads assigned per million genome-mapped read (RPM) ≥1000 in humans or monkeys are depicted. Source data of **b**–**c** are provided in the Source Data file

Notably, <30% of the os-piRNA clusters overlapped with the human testis-piRNA clusters (Fig. 5a, b), proving that oocyte and testes piRNAs are two different classes of piRNAs with different functions and that their expression appears to be regulated by distinct transcription factors. In contrast, more than 97% of the os-piRNA clusters and the 30-nt oocyte piRNA clusters overlapped, and these clusters served as hotspots for producing 94% of the os-piRNAs (Supplementary data 7). The expression levels of the os-piRNAs and the 30-nt oocyte piRNAs complementary to the same transposon were significantly correlated (Supplementary Fig. 6e, f), which may generate additive effects to silence transposons. A significant proportion of os-piRNAs and 30-nt piRNAs from the same locus shared the same 5′ end (Supplementary Fig. 7a, b), whereas no significant correlation was observed between their expression levels (Supplementary Fig. 7c, *R* = 0.12), implying that different mechanisms may be involved in their processing. We also found that the 10-nt 5′-end overlap signature in os-piRNAs was much higher than that between the os-piRNAs and the 30-nt piRNAs (Supplementary Fig. 7d, e). This finding proves that ping-pong processing in os-piRNAs is mainly homotypic and is possibly mediated by HIWI3, and that the heterotypic ping-pong processing between the os-piRNAs and the 30-nt piRNAs is rare in human oocytes.

To further investigate the expression of small RNAs in other primates, we sequenced small RNAs in the oocytes of *Macaca fascicularis*, which are known as crab-eating monkeys. The compositions of small RNAs expressed in the oocytes of monkeys and humans were similar. os-piRNAs were abundantly expressed in monkey oocytes and had similar features to human os-piRNAs, including the 1U/10A preference and the 5′-end 10-nt overlap signature, as well as the single-strand bias (Supplementary Fig. 8a–h). The average length of monkey os-piRNAs were 1 nt shorter than that in humans, which may be due to a slight difference in the footprint of os-piRNA-binding proteins between species. The highly expressed os-piRNA clusters in humans and monkeys were conserved in the genome (Fig. 5c, Supplementary Data 8–9), implying that os-piRNA might have similar functional roles in primate female germ cells.

### Profiling small RNAs in early human embryos

**Fig. 6:**
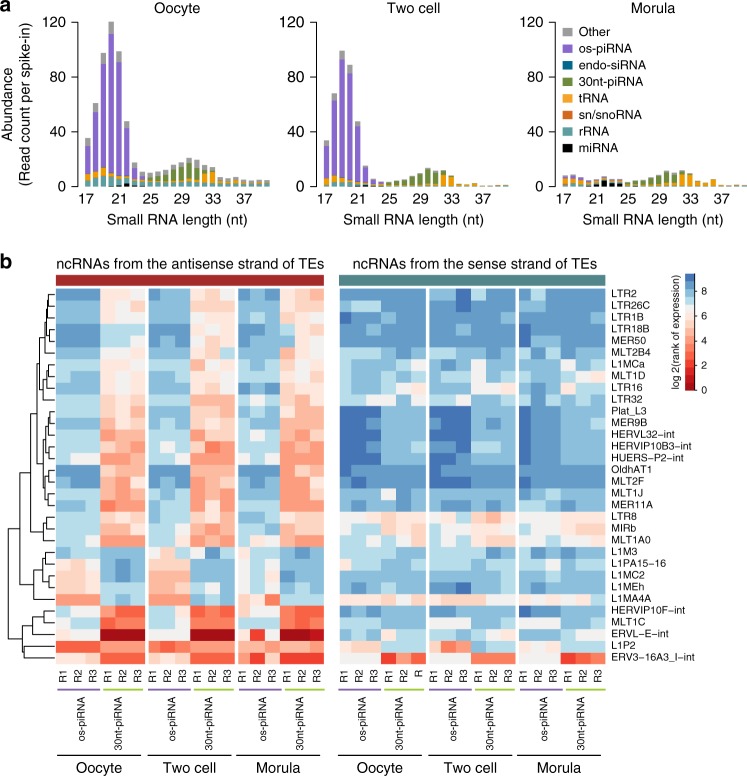
Expression of small RNAs in human early embryos. **a** Abundance and length distribution of small RNAs in human oocytes and early embryos. The expression of small RNAs was normalized to an exogenous spike-in. **b** The highly expressed transposable elements (TEs) that were differentially regulated by oocyte short-PIWI-interacting RNAs (os-piRNAs) and the 30-nt piRNAs in human oocytes are shown. The TEs were ranked in descending order based on the relative abundance of their corresponding piRNAs. The expression ranks of os-piRNAs and the 30-nt piRNAs from the antisense strand of each TE were analyzed by a *t* test (false discovery rate (FDR) ≤0.05). Only the top 50 TEs that were targeted by os-piRNAs or the 30-nt piRNAs were included. Source data of **a**, **b** are provided in the Source Data file

**Fig. 7:**
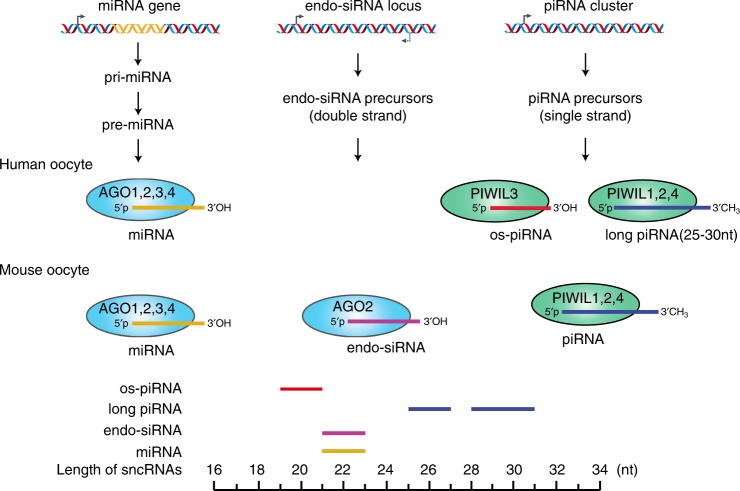
Comparison of the Argonaute family proteins associated with small RNAs in human and mouse oocytes. MicroRNA (miRNA), endo-small interfering RNA (endo-siRNA), and PIWI-interacting RNAs (piRNA) are the three types of small RNAs that bind to Argonaute family proteins. Uracil (U) is the preferred first nucleotide for all the types of piRNAs, and adenine (A) is the preferred tenth nucleotide. Compared to the other piRNAs in oocytes, the HIWI3-associated 20-nt oocyte short piRNAs (os-piRNAs) are shorter and do not bear the 2′-O-methylated modifications at the 3′ terminus. The expression of long piRNAs (≥25 nt) was relatively lower than the expression of os-piRNAs (~20 nt) in human oocytes and the expression of endo-siRNAs (~22 nt) in mouse oocytes

We further sequenced small RNAs from in vitro cultured human oocytes and from early embryos with synthetic short RNA oligos added as spike-ins for an abundance comparison (Fig. 6a). In contrast to the simultaneous degradation of endo-siRNAs and piRNAs in early embryos of mice^28,32^, these 20-nt os-piRNAs declined more rapidly than the 30-nt piRNAs in human morula embryos (Fig. 6a) and correlated with the disappearance of HIWI3 mRNA during human embryo development (Supplementary Fig. 4a)^40^. Comparing the expression of os-piRNAs and the 30-nt piRNAs derived from each TE, we found that os-piRNAs were more prone to target TE members belonging to the L1 family than other targets (Fig. 6b, Supplementary Data 10). In contrast, the 30-nt piRNAs preferred to target ERV and LTR family members, including the most highly expressed ERVL-E-int, indicating that these two classes of piRNAs may have different functions in silencing TEs. The average length of the os-piRNAs in the two-cell and morula stages became ~1–2 nt shorter than those in oocytes, suggesting that 3′ end trimming contributes to os-piRNA degradation. The rapid degradation of os-piRNAs in early embryos supports the idea that their function may be mainly confined to human oogenesis and that they need to be eliminated promptly in early embryos. In contrast, the 30-nt oocyte piRNAs maintained a relatively stable level in the two-cell and later stages, implying that they may play specific roles in genome reprogramming during early embryonic development, warranting further investigation. All of these results support that these two kinds of piRNAs in primate oocytes bind to different piRNA family members and might have distinct processing and turnover pathways (Fig. 7).

## Discussion

In this report, we profiled small RNAs in primate oocytes and early embryos and identified a class of 20-nt piRNAs associated with HIWI3 in human oocytes, namely, os-piRNAs. These os-piRNAs are highly expressed in human oocytes but have not been identified by previous studies^33,58^, possibly because the abundance of oocytes is extremely low when compared to that of other cell types (such as cumulus cells, granulosa cells, theca cells, luteal cells, stroma cells, and ovarian surface epithelial cells), which are abundant in ovarian tissues. This phenomenon renders it impossible to detect os-piRNAs in the oocytes by classic sequencing methods using a bulk of total RNA from human ovary tissue. Although the 30-nt piRNAs bearing 2′-O-methylation at the 3′ end can be enriched and detected in the ovaries by treating total RNA with NaIO_4_, the 20-nt os-piRNAs lacking 2′-O-methylation at the 3′ end are completely eliminated by NaIO_4_ treatment. Such a method cannot distinguish between whether the piRNAs bearing the 3′ end 2′-O-methylation are strictly expressed in oocytes or if they are also present in other cells in the ovaries. Therefore, the single-cell small RNA-seq method provides an advantage in the study of small RNAs in rarer cell types, such as mammalian oocytes. To improve the sensitivity of small RNA-seq, especially for detecting small RNAs expressed at a low abundance, we have developed CAS-seq, which employs Cas9-sgRNA to reduce adapter heterodimers and IVT linear amplification to suppress PCR bias during amplification. Compared to a previous single-cell small RNA-seq method^59^, CAS-seq provides a significantly higher genome-mapping ratio and can reliably detect hundreds to thousands of small RNAs expressed at low levels, such as piRNAs and endo-siRNAs. CAS-seq strictly relies on the presence of NGG (PAM sequence for spCas9/sgRNA recognition and cleavage) in the 3′ adapter. Fortunately, the most frequently used protocols for small RNA-seq, such as TruSeq (Illumina, USA), use a 3′ adapter started with the TGG, which is the same method we utilized in CAS-seq. Other Cas family proteins recognizing different PAM sequences may be used to circumvent the NGG sequence limitations for custom-designed adapters. In addition, the cleavage of RNA/DNA hybrid substrates by Cas9 also extends the substrate spectrum of spCas9, which could also be easily adapted for use in other applications.

The os-piRNAs in human oocytes were approximately 20 nt in length, at least 6 nt shorter than the known piRNAs in the testes and ovaries of other species, and might be processed from a primary transcript by an unknown single-strand-specific endonuclease at 20-nt cleavage intervals^42,46–48^. A recent structural study of the Siwi protein in complex with piRNAs provided valuable insights into the piRNA-PIWI protein interaction, and solving the structure of the HIWI3-piRNA complex might provide structural insight into why the HIWI3-associated piRNAs are much shorter than the piRNAs associated with other PIWI proteins in mammals. The AGO protein family can be divided into the AGO subfamily and the PIWI subfamily^60^. The AGO subfamily is associated with 20–23-nt miRNAs and endo-siRNA, while the PIWI subfamily is associated with 26–30-nt piRNAs. Most mammals encode PIWIL1–4, but the mouse genome lacks PIWIL3. Instead, an oocyte-specific Dicer isoform that results from retro-transposon insertion is highly expressed in mouse oocytes and can produce endo-siRNAs more efficiently^61^. This difference explains the species-specific composition of small RNAs in oocytes: human oocytes express os-piRNAs, long piRNAs, and miRNAs, but lack endo-siRNAs. In contrast, mouse oocytes express endo-siRNAs, long piRNAs, and miRNAs, but lack os-piRNAs (Fig. 7). Previous studies have shown that deficiencies in the piRNA pathways led to female sterility in zebrafish^62^, but had no effect on oogenesis and embryo development in mice^25^. This is possibly due to the presence of abundant endo-siRNAs in mouse oocytes, which are able to compensate for the function of piRNAs in silencing TEs, suggesting that the mouse model is insufficient for the function study of piRNAs in human oogenesis and embryogenesis.

Although the genomic location and the 1U/10A feature of os-piRNAs were similar to that of long piRNAs in human oocytes, the os-piRNA length and tailing nucleotides were more similar to the endo-siRNAs in mouse oocytes. More importantly, we found that the os-piRNAs lack 2′-O-methylation, which is similar to endo-siRNAs that bind to AGO2. Interestingly, in mouse oocytes, MILI was reported to be able to associate with a class of 20–23-nt piRNAs with the non-canonical features of 19 nt complementarity and a 2–4-nt 3′ overhang^33,58^. These findings blur the differences in the sequence features and functions between AGO- and PIWI-associated small RNAs and provide an interesting opportunity to study the adaptive evolution in the mammalian reproduction system. Notably, many oocyte piRNAs were derived from poorly annotated genomic regions. It is conceivable that in addition to silencing TEs, os-piRNAs may have additional functions in global gene expression regulation by targeting numerous transcripts during oocyte development, which warrants future investigation.

## Methods

### Ethics statement

The study has been approved by the Reproductive Study Ethics Committee of Shanghai Ninth Hospital (Research license 20161206). The informed consent process for the embryos and gametes donated complied with the clinical protocols of Department of Assisted Reproduction, Shanghai Ninth People’s Hospital Affiliated to Shanghai Jiao Tong University School of Medicine. The study of human testis has been approved by the Reproductive Study Ethics Committee of China National Population and Family Planning Key Laboratory of Contraceptive Drugs and Devices, Shanghai Institute of Planned Parenthood Research (SIPPR). The informed consent process for the testis donated complied with the clinical protocols of China National Population and Family Planning Key Laboratory of Contraceptive Drugs and Devices, SIPPR.

### Human testes tissue collection

The tissues from the testicular puncture of patients with obstructive azoospermia were quickly frozen on dry ice. The testis samples were stored at −80 °C before RNA extraction.

### Collection of human oocytes, early embryos, and cumulus cells

Ovarian stimulation was conducted as previously described^63^. Briefly, patients at reproductive age were administered human menopausal gonadotropin (150–225 IU; Anhui Fengyuan Pharmaceutical Co.) and medroxyprogesterone acetate (10 mg/day) from MC3 onward. The initiating dose of 150 IU/day was used for patients with high AFC counts that were >20 or slightly elevated basal follicle-stimulating hormone (7–10 IU/L), while 225 IU/day was used for all other patients. Follicular monitoring started on MC7–8 and was performed every 2–4 days using a transvaginal ultrasound examination to record the number of developing follicles. When the diameter of the dominant follicle reached 20 mm, or at least three follicles’ diameter reached 18 mm, the final stage of oocyte maturation was co-triggered by subcutaneous injections of triptorelin (0.1 mg) and human chorionic gonadotropin (hCG) (1000 IU; Lizhu Pharmaceutical Trading Co.). Oocyte pick-up was performed 34–36 h later. After retrieving oocytes and removing cumulus cells, the pre-matured oocytes (GV or MI stage) donated by patients in the cycle of intracytoplasmic sperm injection (ICSI) were cultured in continuous single culture (Irvine Scientific, CA, USA) medium with 10% serum substitute supplement (Irvine Scientific, CA, USA) at 37 °C in an atmosphere of 5% CO_2_. Overnight incubation was performed to develop the mature oocytes (MII) that were inspected under a microscope for clear first polar body formation. Frozen embryos at two-cell and morula stages were donated from couples who have had a baby via ICSI treatment. Cumulus-free high-quality MII oocytes and frozen-thawed embryos were visually inspected under a microscope after all cumulus cells were carefully removed. They were then washed three times by modified HTF medium (Irvine Scientific, CA, USA), and an individual oocytes or embryos were immediately transferred to a PCR tube containing 3 μl diethyl pyrocarbonate (DEPC)-treated water with 6 U of RiboLock RNase inhibitor (Thermo Fisher Scientific, MA, USA) and quickly frozen at −80 °C.

After fertilization via ICSI, embryos were examined for the number and regularity of blastomeres and the degree of embryonic fragmentation on the second day (for two-cell and four-cell stage) and third or fourth day (for eight-cell and morula stage), according to the Cummins et al.’s criteria^64^. High-quality embryos (including grade 1 and grade 2 embryos) were frozen by vitrification either after 1.5 days (for two-cell embryos) or after the fourth day (for morula embryo) post-oocyte retrieval. The vitrification procedure for freezing cleavage-stage embryos was performed using the Cryotop carrier system (Kitazato Biopharma Co.). Embryos were equilibrated in 7.5% (v/v) ethylene glycol and 7.5% (v/v) dimethyl sulfoxide for 5–10 min and then vitrified in a mixture of 15% (v/v) ethylene glycol, 15% (v/v) dimethyl sulfoxide, and 0.58 M sucrose within 1 min. For thawing, solutions of 1, 0.5, and 0 M sucrose were used sequentially as cryoprotectant dilutions for 1n, 3, and 5 min. All vitrification and warming steps were carried out at room temperature except for the first warming step which was conducted at 37 °C.

In order to study the influence of culture time on the small RNA profiling of human oocytes, freshly acquired GV oocytes were cultured for 2–4 or 23–25 h after cumulus cells were removed. All GV stage oocytes were inspected under a microscope to verify GV morphology and the normal ones were transferred to PCR tubes individually as we previously described. To investigate whether constructing small RNA libraries directly using the cell lysis of single oocyte would cause ligation bias or degradation of small RNAs during cell lysis and heat incubation, 5 to 10 GV stage human oocytes were pooled for RNA extraction and approximately 1 ng of total RNA was used for CAS-seq.

### Animal use and care

Animal procedures were conducted in compliance with the ethical guidelines of the Shanghai Institute of Biochemistry and Cell Biology and received ethical approval from the Shanghai Institute of Biochemistry and Cell Biology for acquiring mouse oocytes and mouse testis. Monkey oocyte collection was conducted in compliance with the ethical guidelines of the Institute of Neuroscience and received ethical approval from the Institute of Neuroscience. Shanghai Institutes for Biological Sciences, Chinese Academy of Sciences.

### Collection of monkey and mice oocytes

Adult female *M. fascicularis* monkeys were super-ovulated via intramuscular injection of 15 IU of rhFSH (recombinant human follitropin alfa, GONAL-F, Merck Serono, Germany) twice daily for 8 days and then with 1000 IU of hCG (Sigma, USA) on day 9. Oocytes were collected by laparoscopic follicular aspiration 36 h after hCG injection. Cumulus cells were removed by adding hyaluronidase (2 mg/ml) to the follicle and gently pipetting. Cumulus-free high-quality oocytes (MII) were visually inspected using a microscope and then cultured in HECM-9 medium at 37 °C in an atmosphere of 5% CO_2_ for approximately 2 h. All oocytes and embryos were washed thrice in cold Dulbecco’s phosphate-buffered saline containing 0.01% polyvinylalcohol, immediately transferred to PCR tubes containing 3 µl of DEPC-treated water with 6 U of RiboLock RNase inhibitor (Thermo Fisher Scientific, Massachusetts, USA), and quickly frozen on dry ice. The samples were stored at −80 °C before use. Female C57BL/6 mice (3–4 weeks old) were super-ovulated via intraperitoneal injection of 10 IU of pregnant mare serum gonadotropin and 10 IU of hCG at 43- to 44-h intervals. Cumulus–oocyte complexes were collected from the oviducts 13 to 15 h later and treated with bovine testicular hyaluronidase (1 mg/ml, Sigma-Aldrich) for 1 to 2 min. Cumulus cells were removed and high-quality oocytes (MII) were then collected.

### RNA extraction and quantification

Total RNAs were extracted from mouse oocytes and cultured cells using TRIzol reagent (Ambion), respectively. The quantity of isolated RNA was measured with Qubit 2.0 Fluorometer (Invitrogen).

### Cell culture

HEK293, HeLa, and A549 cells were obtained from ATCC (Manassas, Virgina, USA) and grown in Dulbecco’s modified Eagle’s medium (GIBCO) supplemented with 10% fetal bovine serum (Gibco). Mycoplasma contamination detection was conducted regularly.

### Cas9/sgRNA *i*n vitro cleavage assay

A 66-nt-long single-strand DNA with 5′ Cy5 label (CY5-ssDNA) was chemically synthesized (Sangon Biotech, Shanghai, China) and served as substrate 1 in Fig. 1c. Substrates 2–4 were prepared by annealing CY5-ssDNA with 1.5-fold molar of 3′ adapter, ssDNA-RC (single-strand DNA with sequence reverse and complementary to ssDNA), and adapter chimeras, respectively. Adapter-chimera oligo was ligation product of 3′ adapter and T7-5′ adapter and was purified by denaturing polyacrylamide gel electrophoresis (PAGE). sgRNA in Fig. 1b was produced by IVT with T7 RNA polymerase (NEB, Massachusetts, USA). Cas9/sgRNA complexes were reconstituted by incubating equal molar of Cas9 (NEB, Massachusetts, USA) and sgRNA in 1× Cas9 reaction buffer (20 mM HEPES, 100 mM NaCl, 5 mM MgCl_2_, 0.1 mM EDTA) (NEB, Massachusetts, USA) at 37 °C for 15 min. The cleavage reactions were carried out with 0.1 pmol Cy5-labelled substrates, 1 pmol reconstituted Cas9/sgRNA, and 2.5 U of RiboLock RNase inhibitor (Thermo Fisher Scientific, Massachusetts, USA) in 1× Cas9 reaction buffer at 37 °C for 1 h. For denatured PAGE analysis, the reactions were quenched by adding equal volume of 2× RNA loading buffer (deionized formamide with 5 mM EDTA, no loading dyes) and boiled at 95 °C for 1 min. Then, samples were cooled on ice before being separated by 12% denaturing PAGE. For native gel analysis in Figure S1a, the Cas9/sgRNA cleavage products were mixed with 1/10 volume of 10× RNA loading buffer (30% glycerin with 5 mM EDTA, no loading dyes) and separated by 12% native PAGE. The Cy5-labelled ssDNAs on the gel were detected by FLA-9000 Starion (Fujifilm Life Science, USA). The oligo sequences of CY5-ssDNA, 3′ adapter, ssDNA-RC, adapter chimera, and DNA template for 5′ adapter-sgRNA used for Cas9/sgRNA cleavage assay were listed in Supplementary Data 11.

### CAS-seq cDNA library preparation

Adapter ligation: A single oocyte in 3 μl of RNase-free water was incubated at 95 °C for 2 min and immediately placed on ice. If 1 ng of purified RNA was used, this step was omitted. Then, the sample was mixed with 0.25 pmol of 3′ adapter, 1 μl of 10× T4 ligase reaction buffer, 2 μl of 50% PEG8000, 1 μl of 80 mM MgCl_2_, 40 U of RNL2tr K227Q (200 U/μl, NEB, Massachusetts, USA), 20 U of RiboLock RNase inhibitor (Thermo Fisher Scientific, Massachusetts, USA) and RNase-free water in a 10 μl reaction volume. The sample was incubated at 22 °C for 2 h, followed by the addition of 1 μl of 5 μM biotin-RT primer before heat inactivation at 75 °C for 5 min. The total ligation product was used for 5′ adapter ligation in a 14.5-μl reaction containing 5 pmol of the 5′ T7 adapter, 0.69 mM of ATP, 10 U of T4 RNA ligase 1 (10 U/μl, NEB, Massachusetts, USA), and 20 U of RiboLock RNase inhibitor (Thermo Fisher Scientific, Massachusetts, USA), which was incubated at 22 °C for 1 h.

First-strand cDNA synthesis: All the ligation products were used as templates for RT in a final 25-μl reaction volume containing 125 U of M-MuLV reverse transcriptase (200 U/μl, NEB, Massachusetts, USA), 1× RT reaction buffer, 10 mM dithiothreitol (DTT), 0.5 mM of each dNTP, and 20 U of RiboLock RNase inhibitor (Thermo Fisher Scientific, Massachusetts, USA). The RT reaction was performed at 44 °C for 1 h.

Cas9/sgRNA treatment: An sgRNA with the indicated sequence (Fig. 1b) was produced by IVT using HiScribe T7 Quick High Yield RNA Synthesis Kit (NEB). Cas9 (M0386S, NEB, Massachusetts, USA) and 5′ adapter-sgRNA were mixed at an equimolar ratio in 1× Cas9 reaction buffer in an appropriate volume (e.g., 10 μl Cas9-sgRNA mix included 1× NEB buffer, 2 pmol Cas9, 60 ng 5′ adapter-sgRNA and water) and incubated at 37 °C for 15 min. Then, 0.2 pmol of preincubated Cas9/sgRNA mixture (1 μl of Cas9/sgRNA mixture prepared above) was added to the RT reaction and incubated at 37 °C for 30 min, followed by the addition of 25 U of RNase If (NEB, Massachusetts, USA) and an incubation at 37 °C for 30 min.

On-bead IVT: A T7-promoter oligo (10 pmol) was added to each tube, and the mixture was heated at 95 °C for 5 min and slowly cooled to room temperature. Dynabeads M-280 streptavidin (Thermo Fisher Scientific, Massachusetts, USA) were prepared according to the manufacturer’s instructions. Fifteen microliters of beads (5 μg/μl) was added to each sample and then rotated for 15 min at room temperature to couple the cDNA and beads. IVT was performed with cDNA on beads in a 20-μl reaction volume containing 1× Transcription Optimized buffer (Promega, Wisconsin, USA), 4 mM of each rNTP, 14.5 mM MgCl_2_ (Sigma, USA), 2.5 pmol of the T7-promoter oligo, 10 mM DTT, 40 U of RiboLock RNase inhibitor (Thermo Fisher Scientific, Massachusetts, USA), 0.08 U of inorganic pyrophosphatase (NEB, Massachusetts, USA), and 16 U of high concentration T7 RNA Polymerase (80 U/μl, Promega, Wisconsin, USA). The mixture was incubated at 37 °C with gentle rotation on a rotator for 15–17 h.

Second RT and PCR amplification: The IVT products attached to the streptavidin beads through the biotin-RT primer were washed once with 0.1× binding buffer (0.5 mM Tris-HCl, pH 7.5; 0.05 mM EDTA; 0.1 M NaCl) on a magnetic separation rack. The IVT RNAs adhered to the beads were used directly as the template for the RT reactions in 20-μl volume containing 100 U of M-MuLV reverse transcriptase (200 U/μl, NEB, Massachusetts, USA), 1× RT reaction buffer, 10 mM DTT, 0.5 mM of each dNTP, and 20 U of RiboLock RNase inhibitor (Thermo Fisher Scientific, Massachusetts, USA) at 44 °C for 1 h on a rotator. The RT product was amplified in a 50-μl reaction volume containing 5 μl of the RT reaction product, 1 U of KOD-Plus-Neo (TOYOBO, Osaka, Japan), 1× PCR buffer, 25 pmol of the RP1 primer, 25 pmol of the RPI primer, 1.5 mM MgSO_4_, 0.2 mM each dNTP, and H_2_O. The PCR mixture was heated to 94 °C for 2 min, followed by the appropriate number (usually 15–18 cycles for 1 ng of input RNA or a single oocyte) of cycles at 98 °C for 10 s, 60 °C for 30 s, and 68 °C for 15 s, with a final cycle at 68 °C for 5 min.

Gel purification: The RT-PCR products were separated by 6% polyacrylamide gel electrophoresis and visualized using GeneGreen dye (TIANGEN, Shanghai, China). The 130–60-bp DNA fragments corresponding to the small RNA fraction were cut out from the gel. The gel slice was crushed, and the DNA was eluted in TE buffer (10 mM Tris (pH 7.5), 1 mM EDTA) overnight with rotation at room temperature. The liquid phase was passed through a 0.45-μm Spin-X column (Corning, New York, USA), followed by precipitation with 10 μg of linear acrylamide, 1/10th volume of 3M sodium acetate (pH 5.2), and 3 volumes of ethanol.

Sequencing: An Illumina HiSeq 2500 or HiSeq X Ten sequencer was used to sequence the small RNA libraries. The sequencing data were deposited in the Gene Expression Omnibus of the National Center for Biotechnology Information (NCBI) under accession number GSE95218.

The oligo sequences of 3′ adapter, biotin-RT primer, 5′ T7 adapter, 5′ adapter-sgRNA, and T7 promoter used for small RNA library construction were listed in Supplementary Data 11.

### CAS-seq-2E cDNA library preparation

The 2E-CAS-seq protocol was modified on the basis of CAS-seq by adding 4 U of 5′ deadenylase (NEB, Massachusetts, USA) and 9 U of RecJ_f_ (NEB, Massachusetts, USA) immediately after 3′ adapter ligation reaction. RecJf is an exonuclease that only digests single-strand DNA oligos with monophosphate at the 5′ end, but not the 3′ adapter bearing 5′ rApp modification, a 5′ deadenylase is used to remove the rApp modification from the 5′ end of the 3′ adapter, which convert the 3′ adapter to be a suitable substrate for RecJf digestion. The reaction mixture was incubated at 30 °C for 30 min followed by at 37 °C for 30 min. Then, the 3′ adapter ligation product was used for 5′ adapter ligation following the same method described in CAS-seq.

### Polyclonal antibody production

Antibodies used in immunoprecipitation were polyclonal antibodies produced in rabbit by GL Biochem (Shanghai, China). The peptides, C-HDLGVNTRQNLDHVKESK and C-TRQDMKHVKDSKTGSEG were chosen as antigens to produce antibodies for HIWI and HIWI3, respectively. The peptide C-GKPEEPSTQRGPAQ was chosen as the antigen to produce an antibody for HILI. The cysteine at the N terminal of the synthetic peptides was artificially added for later coupling. The rabbits used for antibody production were immunized with antigen for seven rounds, after which bleedings were conducted. Antibodies were then purified by peptide affinity chromatography.

### Immunoprecipitation assay on HEK293 cells

HEK293 cells were transiently transfected with Flag-tagged HILI, HIWI, HIWI2, and HIWI3 plasmid, respectively, through Lipofectamine 2000 (Invitrogen, USA) following standard procedures. The cells were harvested 48 h after transfection and were homogenized in lysis buffer (50 mM Tris-HCl (pH 7.4), 1% NP-40, 150 mM NaCl, 0.5 mM DTT, 1× proteinase Inhibitor cocktail (Sigma, USA)) for 15 min at 4 °C. The extract was centrifuged at 12,000 × *g* for 10 min at 4 °C. Four hundred microliters of supernatant (40 μl saved as input) was mixed with 5 μg antibody specifically recognizing HIWI, HILI, or HIWI3 coupled with Protein G beads and incubated overnight with gentle rotation at 4 °C. After washing the beads with wash buffer composed of 50 mM Tris-HCl (pH 7.4), 0.005% NP-40, and 150 mM NaCl four times, IP pellets were boiled at 95 °C for 5 min with 1× protein loading. Anti-Flag-HRP (Sigma, USA, 1:5000) was used to detect the target proteins through Western blotting. Uncropped immunoblots are provided in the Source Data file.

### RNA-binding protein immunoprecipitation

Protein G Dynabeads (Thermo Fisher Scientific, Massachusetts, USA) were washed twice with RNA-binding protein immunoprecipitation (RIP) lysis buffer composed of 50 mM Tris-HCl (pH 7.4), 150 mM NaCl, 1 mM EDTA, 0.5% NP-40, 0.5 mM DTT, and 0.4 U/μl of proteinase inhibitor cocktail (Sigma, USA). Two micrograms of antibody and 10 μl original bead mixture were incubated in 100 μl RIP lysis buffer on a rotator at room temperature for half an hour. Antibody-coupled beads were washed twice with RIP buffer before use. Approximately 20 mg of human testis tissue was ground in liquid nitrogen and was lysed in 1.1 ml cold RIP lysis buffer in a protein low-binding tube (Eppendorf, Germany) on ice for 15 min, followed by centrifugation at 16,000 × *g* for 15 min at 4 °C. Each 200 μl testis lysate was transferred into a DNA low-binding tube that contained the antibody-coupled beads for the RIP assay. The remaining 80 μl testis lysate was saved as an input sample. After immunoprecipitation for 1.5 h at room temperature, the beads were washed three times with RIP buffer. The beads were mixed with 30 μl DEPC water, heated at 95 °C for 5 min, and then cooled on ice immediately. The supernatant containing the immunoprecipitated RNA was collected for CAS-seq library construction. The input sample was used for RNA extraction with 600 μl TRIzol Reagent (Ambion, USA). The RNA was precipitated with 10 μg linear acrylamide in cold ethanol and was resolved in 20 μl DEPC water. A Nanodrop-1000 (Thermo Fisher Scientific, Massachusetts, USA) was used to measure the concentration of the input RNA. Ten nanograms of RNA with 0.25 pmol 3′ adapter and 1 × 10^−9^ pmol of spike-in oligos were used for library construction. Three microliters of supernatant containing the immunoprecipitated RNA was mixed with 0.25 pmol 3′ adapter and 1 × 10^−9^ pmol spike-in oligos for library construction. The CAS-seq protocol for the RIP sample was modified by adding 4 U of 5′ deadenylase (NEB, Massachusetts, USA) and 9 U of RecJ_f_ (NEB, Massachusetts, USA) immediately after the 3′ adapter ligation reaction, and the mixture was incubated at 30 °C for 30 min and then followed by at 37 °C for 30 min.

### RIP on human oocyte

Protein G Dynabeads (Thermo Fisher Scientific, Massachusetts, USA) were washed twice with RIP lysis buffer composed of 50 mM Tris-HCl (pH 7.4), 150 mM NaCl, 1 mM EDTA, 0.5% NP-40, 0.5 mM DTT, 0.1 U/μl of RNase inhibitor (Thermo Fisher Scientific, Massachusetts, USA), and 0.4 U/μl of Proteinase inhibitor cocktail (Sigma, USA). Each 0.5 μg of the indicated antibody and 2.5 μl of original beads slurry were incubated in 20 μl RIP lysis buffer on a rotator at room temperature for 30 min. Coupled beads were washed with RIP buffer before use. A total of 31 human oocytes were pooled and lysed by adding 200 μl cold RIP lysis buffer in a low-protein binding tube (Eppendorf, Germany), followed by 16,000 × *g* centrifuging for 5 min at 4 °C. Each 20 μl of oocyte lysis was transferred into a PCR tube that contained the antibody-coupled beads for the RIP assay. After immunoprecipitation for 1.5 h at room temperature, the beads were washed twice with high salt phosphate-buffered saline (PBS with 2 M NaCl), followed by another wash with PBS. The beads were mixed with 5 μl DEPC water containing 5 × 10^−11^ pmol spike-in and 0.25 pmol 3′ adapter, heated at 95 °C for 5 min, and snap-cooled on ice. The supernatant containing immunoprecipitated RNA was collected for CAS-seq library construction. The input and sample supernatants after RIP were mixed with 2 × 10^−9^ pmol spike-in RNA oligos before RNA extraction with 600 μl TRIzol (Ambion, USA). The RNAs were precipitated with 12.5 μg linear acrylamide and resolved in 5 μl DEPC water containing 0.25 pmol 3′ adapter. The CAS-seq protocol for the RIP sample was modified by adding 4 U of 5′ deadenylase (NEB, Massachusetts, USA) and 9 U of RecJ_f_ (NEB, Massachusetts, USA) immediately after the 3′ adapter ligation reaction, and the mixture was incubated at 30 °C for 30 min, then followed by 30 min at 37 °C.

### Antibody information

The source and catalog number of the antibodies we used were as follows: rabbit IgG (Santa Cruz, SC2027); rabbit polyclonal anti-AGO1 (MBL, RN028PW); rat monoclonal anti-AGO2 (Sigma, SAB4200085); mouse monoclonal anti-AGO3 (Active Motif, 39787); mouse monoclonal anti-AGO4 (Merck Millipore, 05-967); mouse monoclonal anti-Flag (Sigma, A8592); rabbit polyclonal anti-GAPDH (glyceraldehyde 3-phosphate dehydrogenase) (Bioworld, AP0063); rabbit polyclonal anti-HIWI (custom-made by GL Biochem); rabbit polyclonal anti-HIWI3 (custom-made by GL Biochem); rabbit polyclonal anti-HILI (custom-made by GL Biochem).

### Spike-in information

Synthesized spike-ins (GenePharma, Shanghai, China) were phosphorylated by T4 Polynucleotide Kinase (Takara, Japan) and pooled, with a final concentration of 10^−9^ μM. For each sample, 1 μl of the spike-in mixture was added before the library construction. There were six spike-ins, and three of them were modified with 2′-O-methylation at their 3′ end. The oligo sequences of spike-ins were listed in Supplementary Data 11, including spike-in 1, spike-in 2, spike-in 3, spike-in 1Me, spike-in 2Me, and spike-in 3Me.

### NaIO_4_ oxidization treatment for small amount of total RNA

For each replicate experiment of oocyte small RNA oxidization, 30 mouse oocytes or 10 human oocytes were used. For the testis RNA, 18.5-day mouse testes were used. The total RNAs were extracted with TRIzol regent according to the manufacturer’s instructions, and then 25 μg linear acrylamide was added and the sample was kept at −30 °C for 30 min before centrifugation. RNAs extracted from 30 mouse oocytes and 10 human oocytes were dissolved in 7 μl DEPC water with 1 × 10^−9^ pmol spike-in and 1 × 10^−8^ pmol spike-in in a DNA low-bind tube (Eppendorf, Germany), respectively. Zero point five microliters of mixture of RNA and spike-in was transferred to a new tube and added with 1 μl of 0.25 μM 3′ adapter and 3.5 μl DEPC water as a no oxidation control. The 6.5 μl mixture of RNA and spike-in was used for oxidization experiment by adding 2 μl of 5× borate buffer (150 mM borax,150 mM boric acid, pH 8.6) and 1.5 μl of freshly dissolved 100 mM NaIO_4_ (Sigma, USA). The reaction mixture was incubated in the dark for 30 min at room temperature and then precipitated using 100% ethanol with 25 μg linear acrylamide and 30 μl sodium acetate (3 M, pH 5.2) and stored at −30 °C for 30 min before centrifugation. The pellet was washed twice with 75% ethanol and dissolved in 5 μl DEPC water containing 0.25 pmol 3′ adapter. Four micrograms and 30 ng mouse testis RNAs were used for NaIO_4_ oxidation as described above, except that the NaIO_4_ concentration for 4 μg testis RNA was 200 mM.

### RT-PCR of small RNAs and expression of miRNAs

In each RNA sample from human cell lines, including HeLa, A549, and HEK293, 1 × 10^−5^ pmol of siEGFP was spiked into each microgram of total RNA. Two micrograms of total RNA were polyadenylated by incubating with 1 mM ATP and 1.25 U of poly(A) polymerase (NEB) at 37 °C for 30 min in a 20 μl reaction system. The polyadenylated RNA was treated with 1 U of deoxyribonuclease I (Thermo Fisher Scientific) at 37 °C for 15 min, and then denatured in the presence of 2.5 mM EDTA at 70 °C for 10 min and snap-cooled on ice. Five hundred nanograms of RNA was reverse transcribed with 200 U of M-MLV Reverse Transcriptase (TAKARA) and 1 μM oligo(dT) according to the manufacturer’s instructions. Real-time PCR was performed with a StepOnePlus Real-Time PCR machine (Applied Biosystems) and SYBR Green reagent (Roche). The amplification conditions were 95 °C for 10 min and 40 cycles of 95 °C for 10 s and 60 °C for 30 s.

Nine miRNAs that have no isoforms (to minimize cross-amplification during RT-PCR) were randomly selected to validate the relative expression levels determined by CAS-seq qRT-PCR (see miRNA candidates and oligos in Supplementary Data 11). miR_x,a_ represents the expression of miRNA x in the cell line a. miR_x,a_/miR_y,a_ represents the relative expression of miR_x_ and miR_y_ in the cell line a. The ratio of the relative expression of miR_x_ and miR_y_ in cell lines a and b was calculated using the equation (cell_a_/cell_b_)_x,y_ = (miR_x,a_/miR_y,a_)/(miR_x,b_/miR_y,b_) and shown in Supplementary Fig. 1f. Pearson’s and Spearman’s correlation were calculated in each plot as well.

### Western blotting of human testis

Twenty milligrams of human testis and human muscle tissues were lysed, respectively, with 1 ml lysis buffer composed of 50 mM Tris-HCl (pH 7.4), 150 mM NaCl, 1 mM EDTA, 0.5% NP-40, 0.5 mM DTT, and 0.4 U/μl of Proteinase inhibitor cocktail (Sigma, USA) on ice for 15 min, followed by centrifugation at 16,000 × *g* for 15 min at 4 °C. Nine hundred microliters of supernatant was transferred to a new tube and used for western blotting with anti-HILI (GL Biochem, 1:1000), anti-HIWI (GL Biochem, 1:1000), anti-HIWI3 (GL Biochem, 1:1000), and anti-GAPDH (Bioworld, 1:2000). Uncropped immunoblots are provided in the Source Data file.

### Mapping of small RNAs

The high-quality reads (17–40 nt) were aligned to the reference genome (hg38 for human, mm10 for mouse, and macFas5 for crab-eating monkey) with Bowtie^65^ allowing no mismatches. The perfectly mapped reads were assigned to known miRNAs, ribosomal RNAs (rRNAs), small nucleolar RNAs (sn/snoRNAs) and transfer RNAs (tRNAs) successively. The reads that could not be mapped to these known small RNAs were used to predict piRNAs, endo-siRNAs, and os-piRNAs successively. For the reads that could be mapped to multiple genomic loci, we used the program reallocate^66^ (http://www.smallrnagroup-mainz.de/software.html) to apportion the read counts of multiply mapped sequences according to their local transcription level calculated by uniquely mapped sequences within 5-kb flanking regions. The reads with low sequence complexity (in which ≥75% of the sequence consists of one nucleotide) were removed from the datasets. The relative abundance of these small RNAs was normalized as reads assigned per million genome-mapped reads (RPMs).

### Annotation of known small RNAs

The miRNA sequences of human and mouse were downloaded from miRBase version 21 (http://www.mirbase.org/). The miRNA sequences with −2, -1, +1, or +2 nt of genome-templated nucleotides at the 3′ ends were annotated as isomiRNAs. The sequences of rRNAs, sn/snoRNAs, and tRNAs were downloaded from Ensembl Genes (www.ensembl.org) and the Genomic tRNA Database (http://lowelab.ucsc.edu/GtRNAdb). For crab-eating monkey sequences, the reads that could be perfectly mapped to the annotated miRNA sequences in miRBase were considered to be miRNAs. The sequences of rRNAs, sn/snoRNAs, and tRNAs predicted by RepeatMasker were downloaded from the genome annotation database of the University of California Santa Cruz (UCSC, http://hgdownload.soe.ucsc.edu/goldenPath/macFas5/database/)

### Prediction of small RNA clusters

The genome sequence was scanned in 5-kb sliding windows with 1-kb increments to identify small RNA clusters that contained at least five small RNA sequences of the same type (at least one of them was uniquely mapped). The cluster boundaries were determined by the small RNA density dropping below 10 small RNAs per kilobase. The length of the clusters was required to be more than 100 bp. Only the small RNA clusters with RPM ≥20 in at least four replicate samples of human/monkey/mouse oocytes were used for further analyses.

### Prediction of piRNAs and piRNA clusters

piRNAs have a preference for U at the 5′ first position (1U) and A at the tenth position (10A) in their nucleotide composition. The piRNA length in mammals is approximately 25–32 nt, and most of them tend to be derived from single-stranded precursors^67^. Therefore, the reads ranging from 25 to 40 nt were used to predict candidate piRNA clusters. The piRNA clusters were required to meet three criteria: (1) more than 75% of the reads were 25–32 nt in length; (2) more than 75% of the reads exhibited the 1U or 10A preference; and (3) more than 75% of reads were derived from the main strand. These analyses were implemented with proTRAC (version 2.1.2)^68^. The command line options were set as follows: -pimin 25 -pimax 32 -pisize 0.75 -1Tor10A 0.75 -clhitsn 2 -clhits 5 -clsize 100 -clstrand 0.75 -dens 10.0 -swsize 5000 -swincr 1000. Reads 25–40 nt in length that could be mapped to these piRNA clusters were predicted as piRNAs.

### Prediction of endo-siRNAs and endo-siRNA clusters

Endo-siRNAs are generated from long double-stranded RNAs or hairpin precursors by Dicer, which generates sequences approximately 21–23 nt in length, and a significant portion can form complementary pairs with a 2-nt 3′ overhang^61^. Compared to piRNAs, endo-siRNAs have no preference for 10A, and the preference for 1U was less significant. Therefore, the reads ranging from 17 to 24 nt in length were used to predict candidate endo-siRNA clusters (see Prediction of small RNA clusters). The endo-siRNA clusters were required to meet two criteria: (1) more than 75% of the read counts were 21–23 nt in length; and (2) at least four sequences in the cluster had a 2-nt overhang, and the ratio of sequences having a 2-nt overhang in the cluster was higher than 10%. Reads 17–24 nt in length that could be mapped to these endo-siRNA clusters were predicted to be endo-siRNAs.

### Prediction of os-piRNAs and os-piRNA clusters

The small RNAs that could neither be mapped to known small RNAs nor predicted to be piRNAs or endo-siRNAs in human oocytes were found to have the following characteristics: a length of approximately 18–22 nt; a preference for 1U and 10A; and an enrichment at 10 nt in the length of the 5′-end overlap between complementary sequence pairs (ping-pong signature). In addition, clusters were observed in the genomic distribution of these small RNAs. Therefore, the remaining reads ranging from 17 to 24 nt in length were used to predict candidate os-piRNA clusters (see Prediction of small RNA clusters). The os-piRNA clusters were required to meet three criteria: (1) more than 75% of the read counts were 18–22 nt in length; (2) more than 75% of read counts exhibited the 1U or 10A preference; and (3) at least four sequences in the cluster had the ping-pong signature, and the ratio of ping-pong sequences in the cluster was higher than 1%. Reads 17–24 nt in length that could be mapped to these os-piRNA clusters were predicted to be os-piRNAs.

### Spike-in normalization

To quantify the expression levels of small RNAs in human oocytes and early embryos, we normalized the read counts of small RNAs to that of spike-ins.

### Analyses of 3′ tailing of small RNAs

Besides the reads that could be perfectly mapped to the human genome, we also collected those with nontemplated nucleotide addition at their 3′ end. The reads that were no shorter than 17 nt and could be perfectly mapped to the os-piRNA/30-nt piRNA clusters after removing 1 to 4 nucleotides from their 3′ end were taken as 3′ tailed ones. The total expression of os-piRNAs/30-nt piRNAs was calculated by adding the count of perfectly matched reads and those with 3′ tailing. The ratio of nontemplated 3′ tailing was defined as the proportion of the 3′ tailed read count over the total read count. Analysis of 3′ tailing of endo-siRNAs and piRNAs in mouse oocytes was performed in the similar way.

### Mapping of small RNAs onto gene elements

The genomic annotation of different gene elements (bed files) was downloaded from the table browser of UCSC (Gencode v23 for human, Gencode VM9 for mouse). Bedtools was used to calculate the ratio of small RNAs derived from each kind of gene elements. The small RNAs were firstly categorized into six classes based on their overlap with the CDS, 5′-UTR, 3′-UTR, intron of protein-coding genes, exon and intron of non-coding genes sequentially, with the priority of sense over antisense, and the remaining ones were considered as intergenic. The genomic distribution of human os-piRNAs was shuffled 100 times with Bedtools, and the average of random results was taken as their random ratio mapping onto each gene element.

### Analysis of piRNA phasing

We followed the method of phasing analysis as described previously by Han et al^42^. Briefly, reads ≥17 nt were mapped to the human genome by Bowtie without allowing any mismatches. The distance from the 5′ end of the upstream piRNA to the 5′ end of the downstream piRNA on the same genomic strand (5′-to-5′ distance) was calculated. For each pair of adjacent reads, the minimum of their read count was taken as the score of their distance *x* nt. For the multiply mapped reads, their expression at each locus was weighted by the number of genomic loci that they could be mapped.

### Mapping of small RNAs onto TEs

The bed files of different repetitive elements predicted by RepeatMasker were downloaded from the genome annotation database of UCSC. In addition, the genomic flanking regions of each repetitive element were also calculated with Bedtools, with FL1k and FL2k representing 0–1- and 1–2-kb flanking region, respectively. The small RNAs were firstly categorized into five classes based on their overlap with LINE, LTR, SINE, DNA, and other repeats, with the priority of sense over antisense. The left ones were further classified into 10 categories, in the order of LINE-FL1k, LTR-FL1k, SINE-FL1k, DNA-FL1k, other repeat-FL1k, LINE-FL2k, LTR-FL2k, SINE-FL2k, DNA-FL2k, and other repeat-FL2k. The other small RNAs remained unclassified were marked as other nonRepeat.

### Conservation of the TE consensus sequences

The consensus sequences and conservation information of transposon consensus sequences were downloaded from Repbase^69^. A maximum mismatch of two nucleotides was allowed when aligning os-piRNAs onto the consensus sequences of transposons with bowtie.

### Identification of differentially targeted TEs

To identify TEs targeted by os-piRNAs and 30-nt piRNAs at post-transcriptional level, we first filtered out the repetitive elements predicted to be rRNA, tRNA, srpRNA, or unknow resource. Since os-piRNAs and 30-nt piRNAs derived from the CDS, 5′-UTR, 3′-UTR, and intron of protein-coding genes showed no antisense preference for TEs, we only used the TEs from non-coding genes or intergenic regions for target analysis. The total expression of os-piRNAs and 30-nt piRNAs on the sense and antisense strand of each TE were calculated separately. A *T* test was utilized to compare the expression rank of os-piRNAs and 30-nt piRNAs derived from each TE, and Bonferroni correction was used to calculate false discovery rate. The TEs with same names may have multiple genomic loci, but the small RNAs mapped to the same TE were only counted once.

### Analyses of DNA methylation

The DNA methylation data of two human oocyte samples^57^ (GSE49828) were download from the NCBI Gene Expression Ominibus. The methylation of cytosines in the context of CpG was defined as CpG methylation, and that in the context of CHH and CHG was defined as non-CpG methylation. The genome coordinates were converted from hg19 to hg38 with LiftOver. Only the cytosines with a read coverage ≥5 were kept for further analyses. The cytosines with a methylation ratio ≥0.25 were considered to be methylated. The methylation ratios of genomic regions, such as os-piRNA clusters, were calculated as the ratio of methylated cytosines to all the cytosines detected by sequencing. The total number of detected cytosines in one region should be no <10. The average methylation ratio in the two replicate samples was used to compare the methylation level of highly expressed os-piRNA clusters (RPM ≥20 in at least four replicate samples) and genes (average fragments per kilobase of transcript per million >0).

### Transcription analyses of os-piRNA clusters

The RNA-seq data of two human oocytes detected by strand-specific library (GSE85632)^40^ and three human oocyte samples detected by non-strand-specific library (GSE36552)^39^ were downloaded from the NCBI Gene Expression Ominibus. The genome coordinates in the downloaded bed files of GSE36552 were converted from hg19 to hg38 with LiftOver. The transcription level of os-piRNA clusters was calculated using Bedtools. After filtering with sequencing quality and removing adapters, the reads from GSE85632 were mapped to human genome (hg38) with hisat2, and the distribution of reads on each strand of os-piRNA clusters were calculated with samtools.

### Conservation analysis of os-piRNA clusters

The homologous relationship between human and monkey os-piRNA clusters and those among human, monkey, and mouse endo-siRNA clusters were calculated using LiftOver.

### Comparison between os-piRNA clusters and piRNA clusters

The bed file of previously reported piRNA clusters in human testis was downloaded from a piRNA cluster database^66^. To compare os-piRNA clusters in human oocytes with piRNA clusters in human oocytes and testes, we merged the genomic locations of these three kinds of small RNA clusters, and then calculated the number of clusters overlapped with each other.

### Annotation of small RNAs in human GV oocytes and RIP assays

os-piRNA/piRNA/endo-siRNA clusters with RPM ≥20 in at least four of the six replicate samples of human oocytes were selected as being oocyte-derived ones. After filtering known miRNAs, rRNAs, sn/snoRNAs, and tRNAs successively, the remaining reads in human GV oocytes or RIP samples were mapped to the clusters of oocyte-derived piRNA, endo-siRNA, and os-piRNA successively. Reads that could be mapped to oocyte-derived piRNA clusters and longer than 24 nt were annotated as piRNAs. Reads 17–24 nt in length that could be mapped to oocyte-derived endo-siRNA and os-piRNA clusters were annotated as endo-siRNAs and os-piRNAs, respectively.

### Annotation of small RNAs in human testes

The piRNA clusters of human testes predicted from previously published data were downloaded from piRNA cluster database^66^. The endo-siRNA and os-piRNA clusters predicted from human oocyte samples were also included. After filtering known miRNAs, rRNAs, sn/snoRNAs, and tRNAs successively, the remaining reads in the total RNA and the RIP of human testis samples were mapped to the clusters of testis-piRNA, oocyte endo-siRNA, and os-piRNA clusters successively.

### Reporting summary

Further information on experimental design is available in the Nature Research Reporting Summary linked to this article.

## Supplementary information

Supplementary Information

Reporting summary

Description of Additional Supplementary Files

Supplementary Data 1

Supplementary Data 2

Supplementary Data 3

Supplementary Data 4

Supplementary Data 5

Supplementary Data 6

Supplementary Data 7

Supplementary Data 8

Supplementary Data 9

Supplementary Data 10

Supplementary Data 11

Source Data

## Data Availability

A reporting summary for this Article is available as a Supplementary Information file. The source data underlying the figures in the manuscript is provided as a Source Data file. The deep sequencing data have been deposited in the National Center for Biotechnology Information Gene Expression Omnibus under accession number GSE95218. All data including the genomic distribution of small RNAs (WIG or Bed file), the os-piRNA complementary pairs with ping-pong signature, and the expression small RNAs derived from sn/snoRNAs, rRNAs and tRNAs is available from the corresponding author upon reasonable request.
