# Supplementary material for: Single-cell CAS-seq reveals a class of short PIWI-interacting RNAs in human oocytes
Source: Nat Commun. 2019 Jul 29;10:3389. doi: 10.1038/s41467-019-11312-8 (PMC6662892; doi:10.1038/s41467-019-11312-8)
Supplement: Supplementary file 1 — Supplementary Information [file 41467_2019_11312_MOESM1_ESM.pdf]

Supplementary Information

Single-cell CAS-seq reveals a class of short PIWI-interacting RNAs in human oocytes

Qiyuan Yang[#], Ronghong Li[#], Qifeng Lyu[#], Li Hou[#], Zhen Liu, Qiang Sun, Miao Liu, Huijuan Shi, Beiying Xu, Mingru Yin, Zhiguang Yan, Ying Huang, Mofang Liu, Yiping Li[*] & Ligang Wu[*]

[#] These authors contributed equally to this work.

[*] Correspondence: yipingli@sibcb.ac.cn (Y.L.); lgwu@sibcb.ac.cn (L.W.)

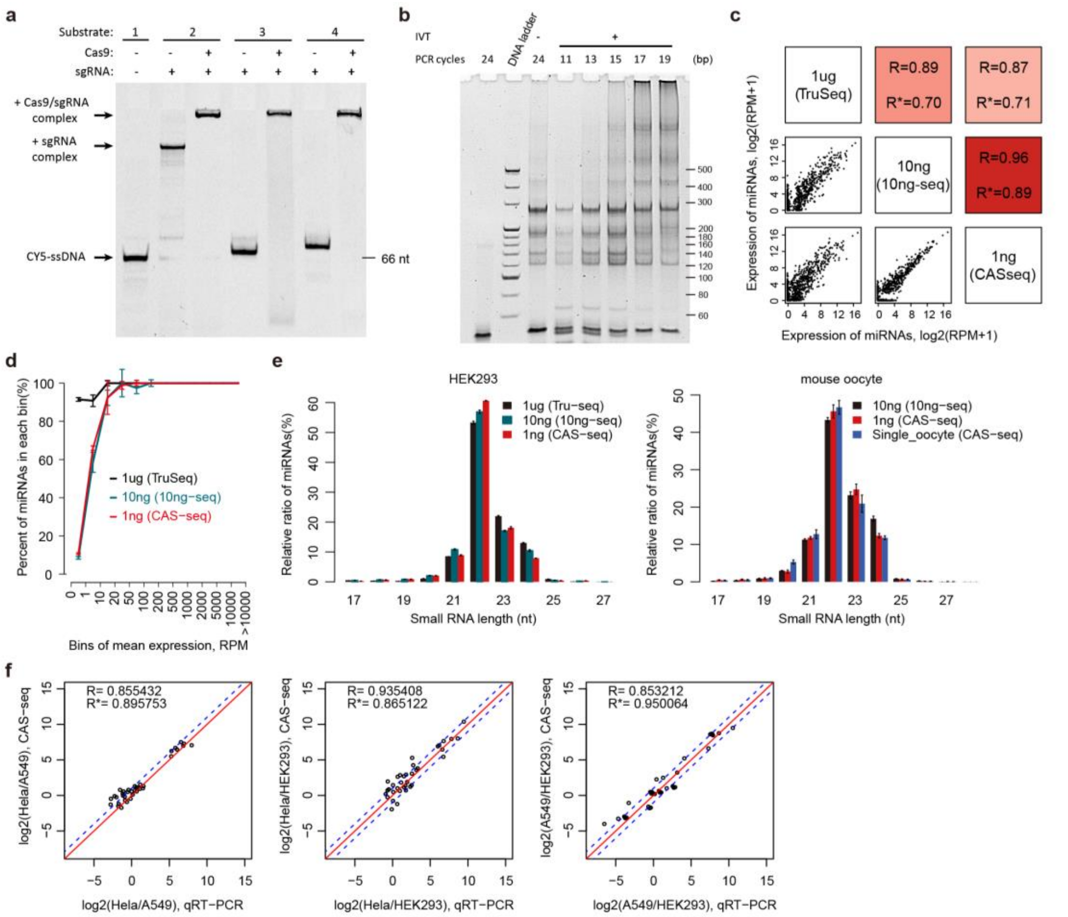

Supplementary Figure 1. Cas9-sgRNA design and the performance of CAS-seq to detect miRNAs

(a) Native PAGE analysis of Cas9-sgRNA cleavage products with substrate illustration in Figure 1c. (b) IVT amplification efficiency was analysed by a series of PCR cycles with an equal amount of input RNA subjected to IVT or non-IVT treatment. (c) Comparison of HEK293 cell small RNA profiling by different approaches, including TruSeq (Illumina) with 1 µg of total RNA, 10 ng-seq with 10 ng of total RNA and CAS-seq with 1 ng of total RNA. Two or three replicates were analysed in each group, and the average expression levels of miRNAs were plotted. (d) The sensitivity of CAS-seq and of other methods to detect miRNAs in HEK293 cells. The mean and S.D. of the percentage of miRNAs detected by each method are shown. (e) The length distribution of miRNAs detected by CAS-seq was similar to that detected by other methods in HEK293 cells or in mouse oocytes. (f) qRT-PCR validation of miRNA expression detected by CAS-seq with 1 ng of total RNA from HEK293, HeLa and A549 cells. R and R* in (c,f) represented Pearson and Spearman

correlation coefficient, respectively. Source data of (a-f) are provided in the Source Data file.

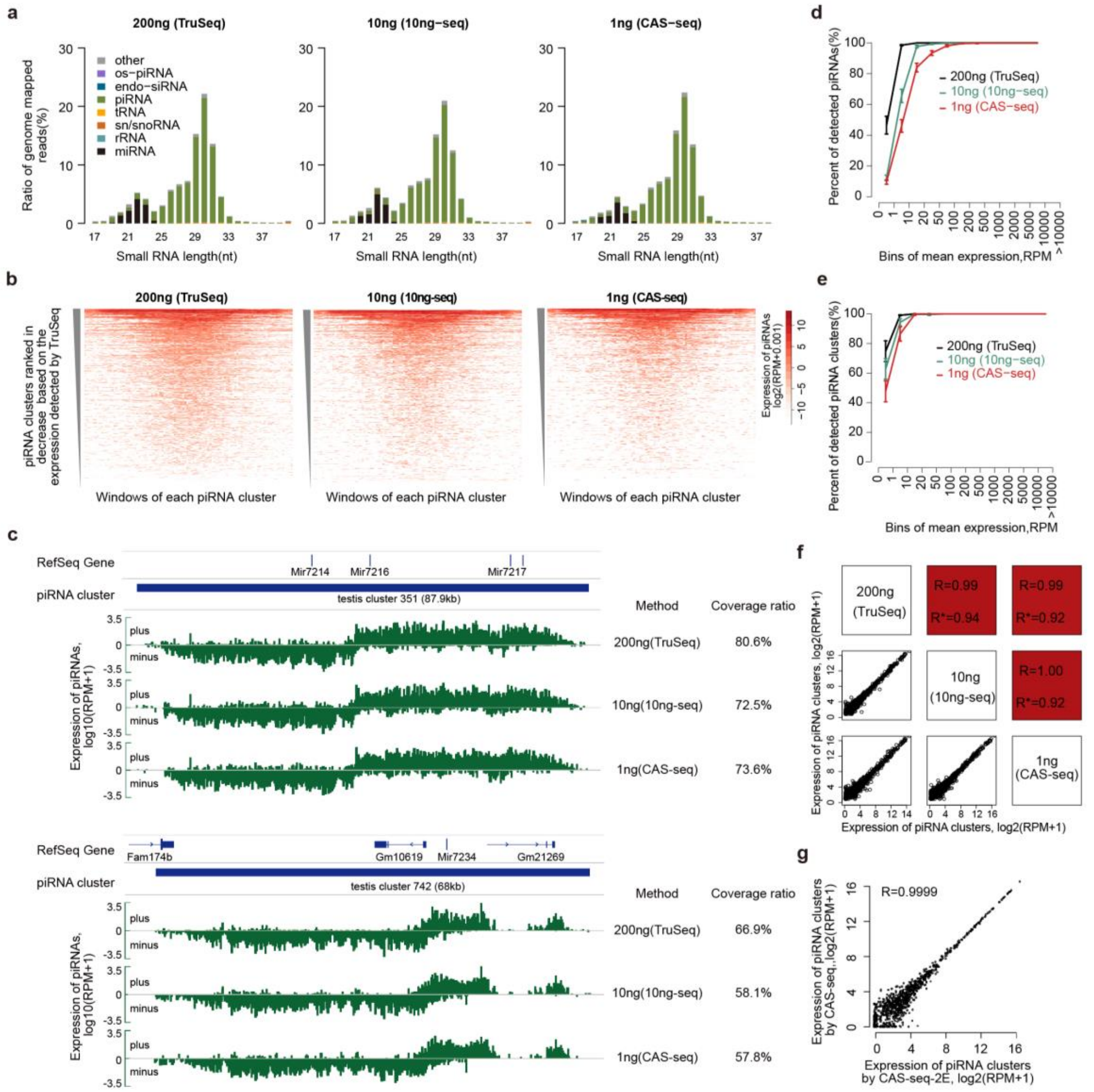

Supplementary Figure 2. The performance of CAS-seq to detect piRNAs

(a) The composition and length distribution of small RNAs in mouse testes detected by CAS-seq were similar to those detected by Illumina TruSeq or 10ng-seq method. (b) The coverage of piRNAs in each piRNA cluster of mouse testes detected by TruSeq, 10ng-seq and CAS-seq. Each piRNA cluster was divided into 100 windows equally in length, and the expression of piRNAs detected in each window was calculated and plotted accordingly. (c) The distribution of piRNAs on the top 2 expressed piRNA clusters in mouse testes. The coverage ratio of piRNAs on each piRNA cluster was calculated. The positive and negative numbers of the vertical axis represent the piRNA expression level on the plus and minus strands, respectively. (d) A sensitivity comparison of the CAS-seq, 10ng-seq, and TruSeq methods to detect piRNAs in mouse testes. (e) A sensitivity comparison of the

CAS-seq, 10ng-seq, and TruSeq methods to detect piRNA clusters in mouse testes. piRNA clusters were classified into 12 groups based on their expression levels detected in 200 ng of total RNA using TruSeq. The mean and standard deviation of the percentage of piRNA clusters detected by each method were shown. (f) The expression of piRNA clusters in mouse testes detected by CAS-seq, 10ng-seq and TruSeq methods were highly correlated. R and R* represented Pearson and Spearman correlation coefficient, respectively. Three replicates were sequenced by each method in (a-f). (g) The expression of piRNA clusters in mouse testes detected by CAS-seq and CAS-seq-2E (CAS-seq with the additional treatment of deadenylase and exonuclease) was highly correlated. R is Pearson coefficient. Three replicate samples were detected by each method. Source data of (a-b, d-g) are provided in the Source Data file.

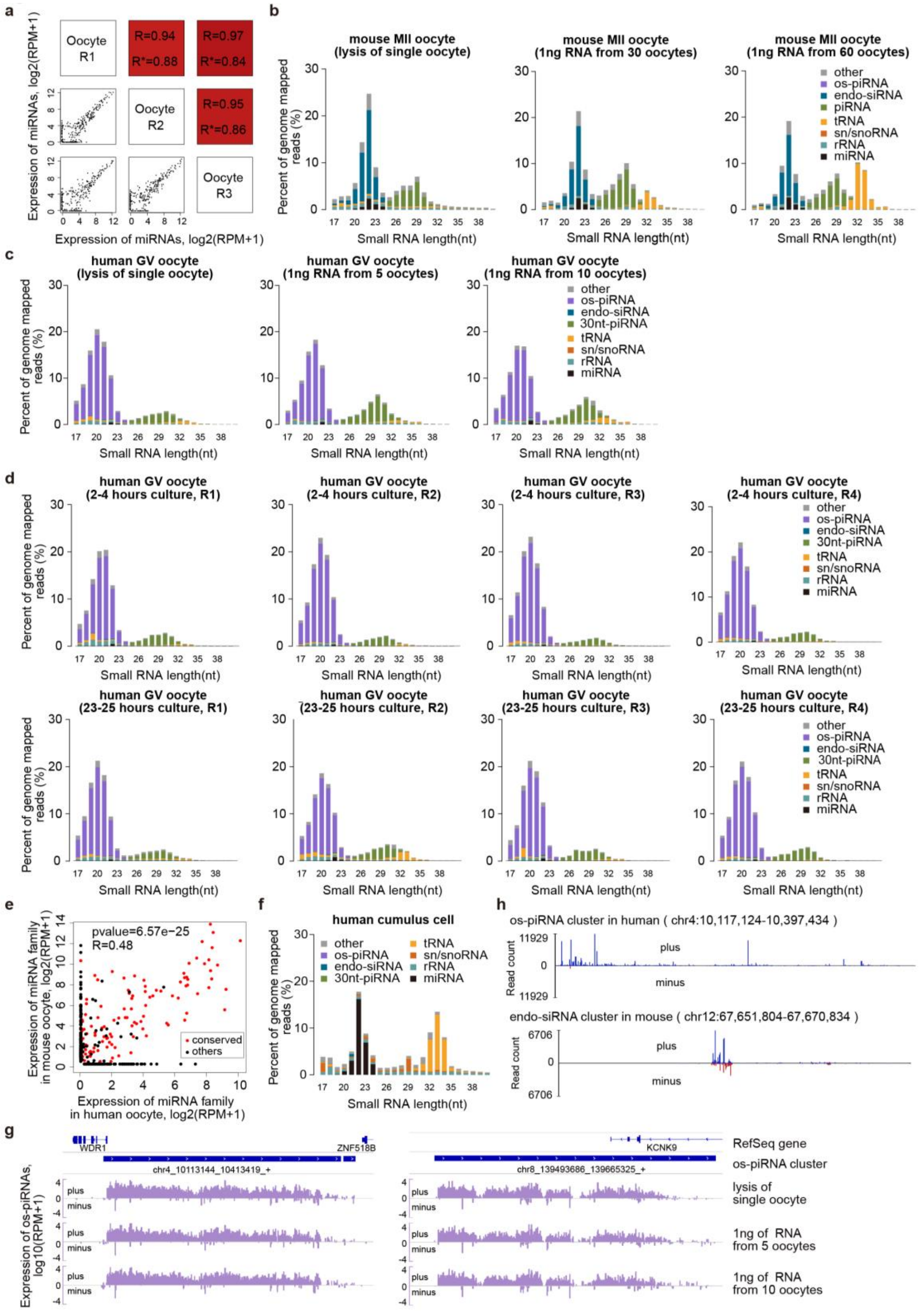

Supplementary Figure 3. Comparison of small RNA profiles in oocytes with different RNA extraction methods and culturing times

(a) Correlation of miRNA profiles in single mouse oocyte detected by CAS-seq. R and R* represented Pearson and Spearman correlation coefficient, respectively. (b) Small RNA

categories according to their length distributions in mouse MII oocytes. The sequencing results of small RNAs using approximately 1 ng of total RNA extracted from 60 or 30 oocytes, or using the lysis of a single oocyte, are shown. (c) Small RNAs are categorized according to their length distributions in human GV oocytes. The sequencing results of small RNAs using approximately 1 ng of total RNA extracted from 10 or 5 GV oocytes or using the lysis of a single GV oocyte are shown. (d) Small RNAs are categorized according to their length distributions in human GV oocytes. Sequencing results of four biological replicates using the lysis of a single GV oocyte cultured for 2-4 hours or 23-25 hours are shown. (e) Correlations of miRNA family expression in human and mouse oocytes. The miRNA families marked with red are those annotated as either conserved or highly conserved by TargetScan[1]. (f) Small RNAs are categorized according to their length distributions in human cumulus cells. (g) The distribution of os-piRNAs on two highly expressed os-piRNA clusters in human GV oocytes of (c). The positive and negative numbers of the vertical axis represent the piRNA expression level on the plus and minus strands, respectively. (h) Illustration of human os-piRNA and mouse endo-siRNA clusters. The small RNAs distributed on the plus and minus strands are shown. Source data of (a-f) are provided in the Source Data file.

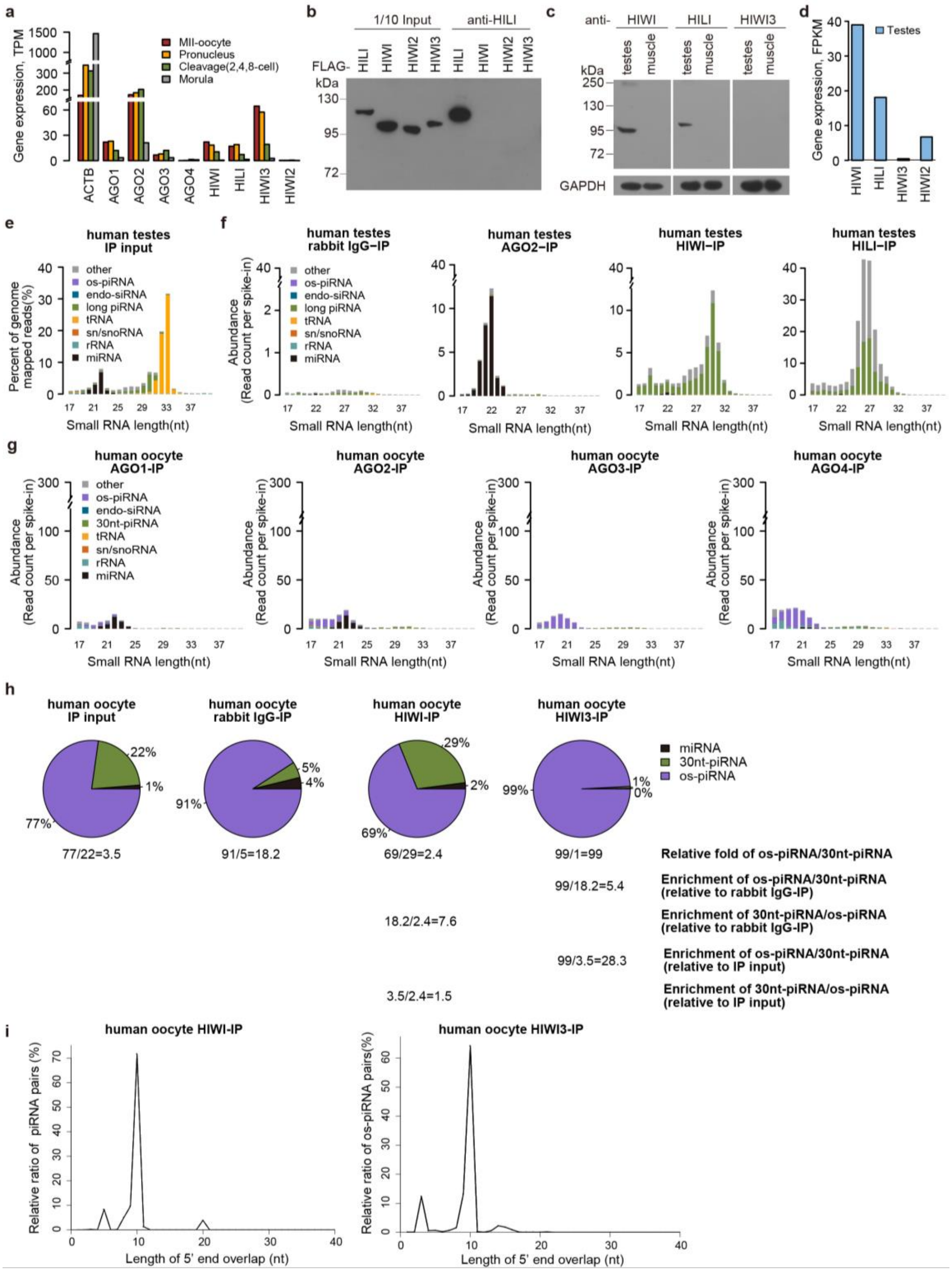

Supplementary Figure 4. Comparison between human oocyte os-piRNAs and other known small RNAs

(a) The relative mRNA abundance of the AGO and PIWI family members in human oocytes and early embryos is shown. (b) The specificity of HILI antibodies is shown. FLAG-tagged HIWI3, HIWI2, HIWI, and HILI were ectopically expressed in HEK293 cells. The cell lysates were immunoprecipitated with HILI-specific antibodies and immunoblotted

with an anti-FLAG antibody. (c) The specificity of HIWI, HILI and HIWI3 antibodies is shown. The lysate of adult human testes was immunoblotted with HIWI, HILI and HIWI3 specific antibodies respectively. The lysate of human muscle served as negative control. (d) The relative mRNA abundance of PIWI family members in the human testes. (e-f) piRNAs with lengths of 26 or 30 nt were enriched by HILI or HIWI immunoprecipitation in human testes, respectively. The abundance and length distribution of small RNAs in the input (e) and IP samples (f) are shown. A rabbit nonspecific IgG antibody served as a negative control. The abundance of small RNAs is normalized to one exogenous spike-in. (g) Abundance and length distribution of small RNAs immunoprecipitated with anti-AGO antibodies. (h) Relative expression level and enrichment fold of miRNAs, os-piRNAs and 30-nt piRNAs in the input, IgG-, HIWI- and HIWI3-immunoprecipitation samples of human oocytes. The ratio of os-piRNAs and 30nt-piRNAs in each sample, the relative enrichment fold of os-piRNAs vs. 30nt-piRNAs in HIWI3 immunoprecipitation, and the relative enrichment fold of 30nt-piRNAs vs. os-piRNAs in HIWI immunoprecipitation over that in IgG immunoprecipitation or over that in oocyte lysate are shown below the graph. (i) Ping-pong signature analyses of the piRNAs associated with HIWI and HIWI3 in human oocytes are shown. Source data of (a-g,i) are provided in the Source Data file.

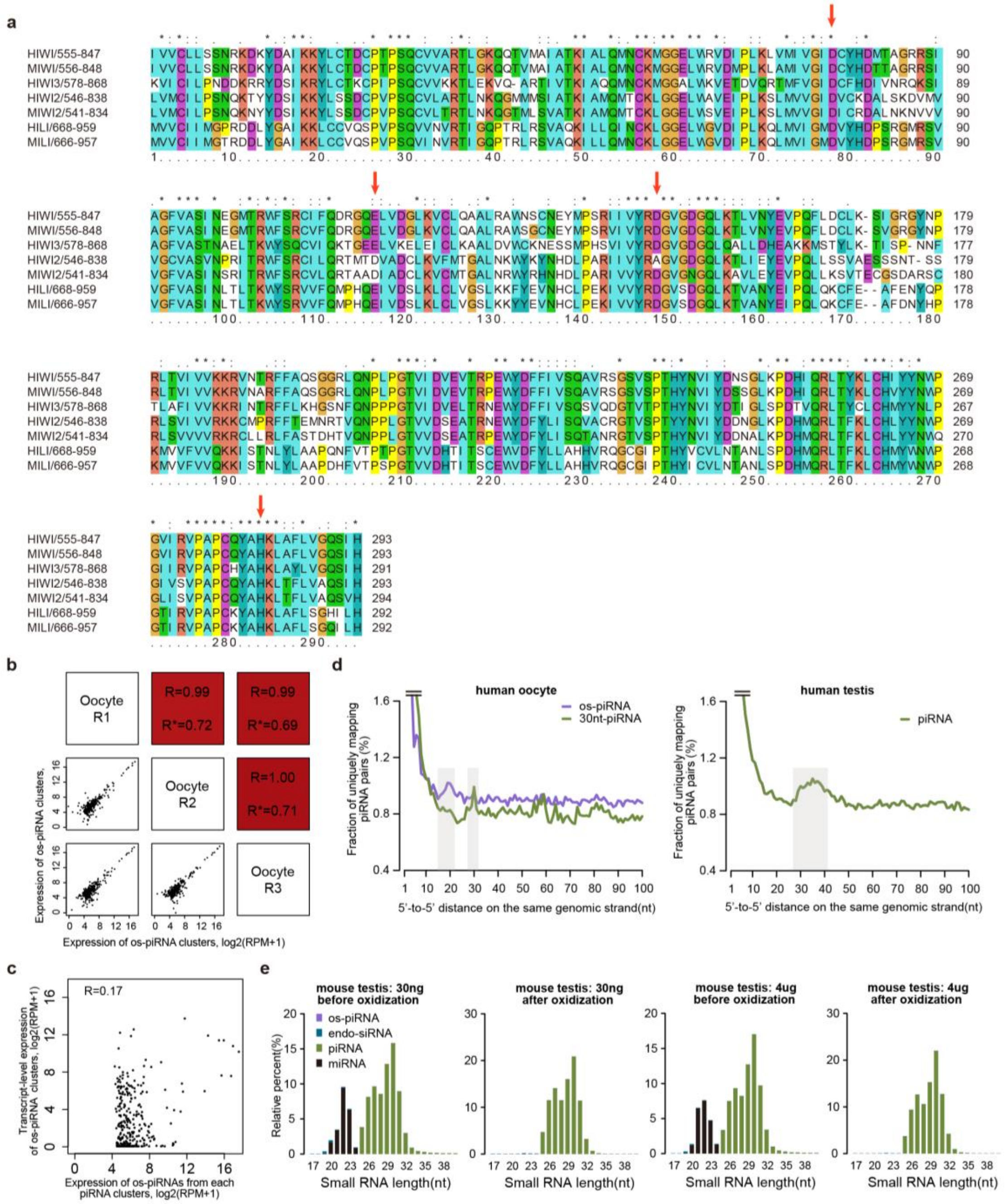

Supplementary Figure 5. Sequence alignment of PIWI family proteins and the features of os-piRNA processing

(a) Alignment of the PIWI domain sequences in PIWI family proteins. The sequence of the PIWI domain in each protein was downloaded from the PROSITE database[2]. The alignment was calculated by ClustalX 2.1[3]. The positions of the catalytic DEDH residues essential for the slicer activity of the HIWI3 protein are indicated by arrowheads. (b) The expression of the os-piRNA clusters in the replicate samples of human oocytes was highly correlated. R and R* represented Pearson and Spearman correlation coefficient,

respectively. (c) The correlation between the os-piRNAs expressed in each cluster and the os-piRNA cluster transcript in human oocytes is shown. (d) Distance from the 5’ end of the upstream piRNAs to the 5’ end of downstream piRNAs on the same genomic strand is depicted. (e) Abundance and length distribution of small RNAs in the mouse testes before and after $NaIO_4$ oxidization are shown. The enrichment fold of piRNAs relative to miRNAs detected from 30 ng RNA was similar to that from 4 μg RNA from mouse testes. The concentrations of $NaIO_4$ used for the 30 ng and 4 μg of RNA were 100 mM and 200 mM, respectively. Source data of (b-e) are provided in the Source Data file.

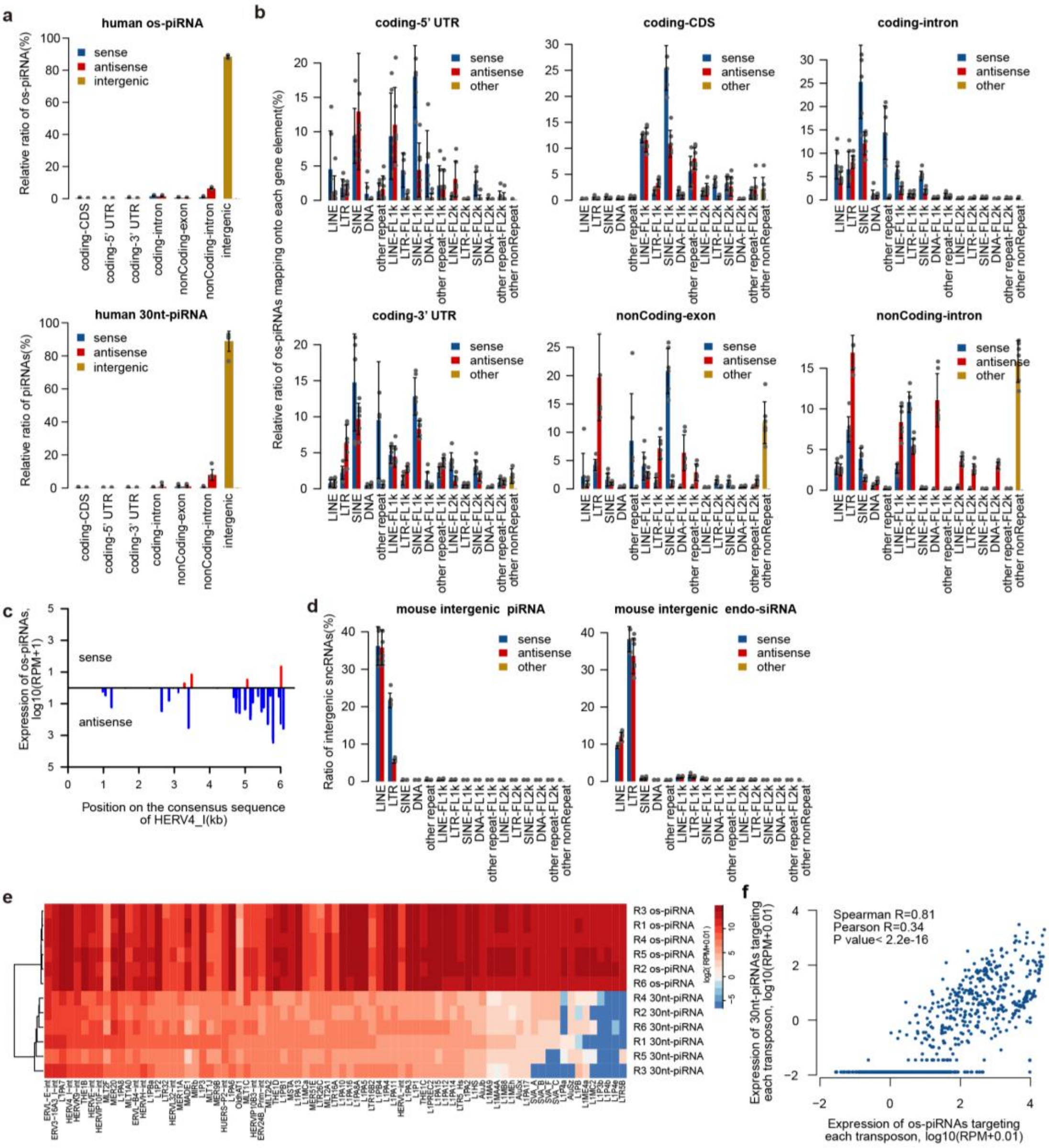

Supplementary Figure 6. Genomic distribution of human os-piRNAs and 30-nt piRNAs

(a) The percentage of human oocyte os-piRNAs and 30-nt piRNAs derived from different gene elements is shown. (b) Proportion of human intergenic os-piRNAs on the sense and the antisense strand of different repetitive elements and their flanking regions. The results of os-piRNAs derived from different gene elements are shown. The average results in six human oocyte replicate samples are shown, and the error bars represent the standard deviation. (c) The distribution of os-piRNAs on the consensus sequence of the highly expressed transposon, HERV4_I, in human oocytes. (d) Proportion of mouse intergenic endo-siRNAs (right) and piRNAs (left) on the sense and antisense strands of different

repetitive elements and their flanking regions. The average results in six mouse oocyte replicate samples are shown, and the error bars represent the standard deviations. (e) The expression heatmap of os-piRNAs and 30-nt piRNAs derived from different human transposons is shown. The top 50 transposons with highly expressed os-piRNAs or 30-nt piRNAs are shown. (f) The expression correlation of os-piRNAs and 30-nt piRNAs targeting different human transposons is shown. Source data of (a-b, d-f) are provided in the Source Data file.

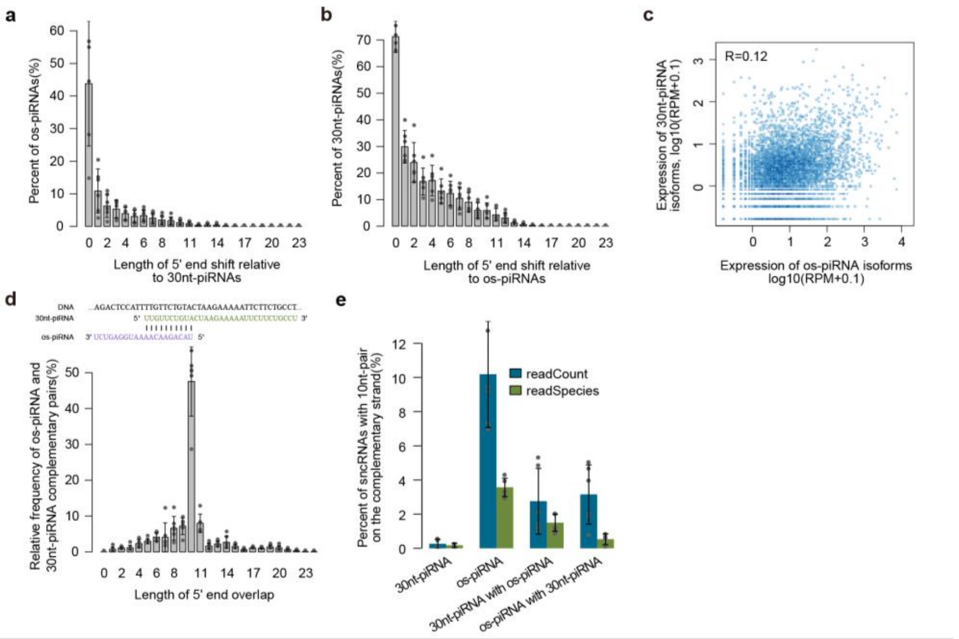

Supplementary Figure 7. Comparison of os-piRNA and 30-nt piRNA sequences

(a) Percent of os-piRNAs having different lengths of the 5’ end shift compared to their 30-nt piRNA homologues. The 30-nt piRNAs containing subsequences similar to the sequences of os-piRNAs were identified as the 30-nt piRNA homologues of os-piRNAs. (b) The percentage of 30-nt piRNAs with short os-piRNA isoforms according to their 5' end shift length is shown. (c) The expression correlation of the os-piRNAs and the 30-nt piRNAs with the same 5’ end in human oocytes is shown. (d) The percentage of partially complementary os-piRNA and 30-nt piRNA pairs with different lengths of 5' overlaps in human oocytes is shown. An example of one os-piRNA paired with a 30-nt piRNA with the 10 nt 5’ overlap signature is shown. (e) The percentage of piRNAs harbouring the 10 nt 5’ overlap signature is shown. The results of human piRNAs are shown as follows: 30-nt piRNAs, os-piRNAs and the combination of these two kinds of piRNAs. The average results from six human oocyte replicate samples are shown, and the error bars represent the standard deviations. Source data of (a-b, d-e) are provided in the Source Data file.

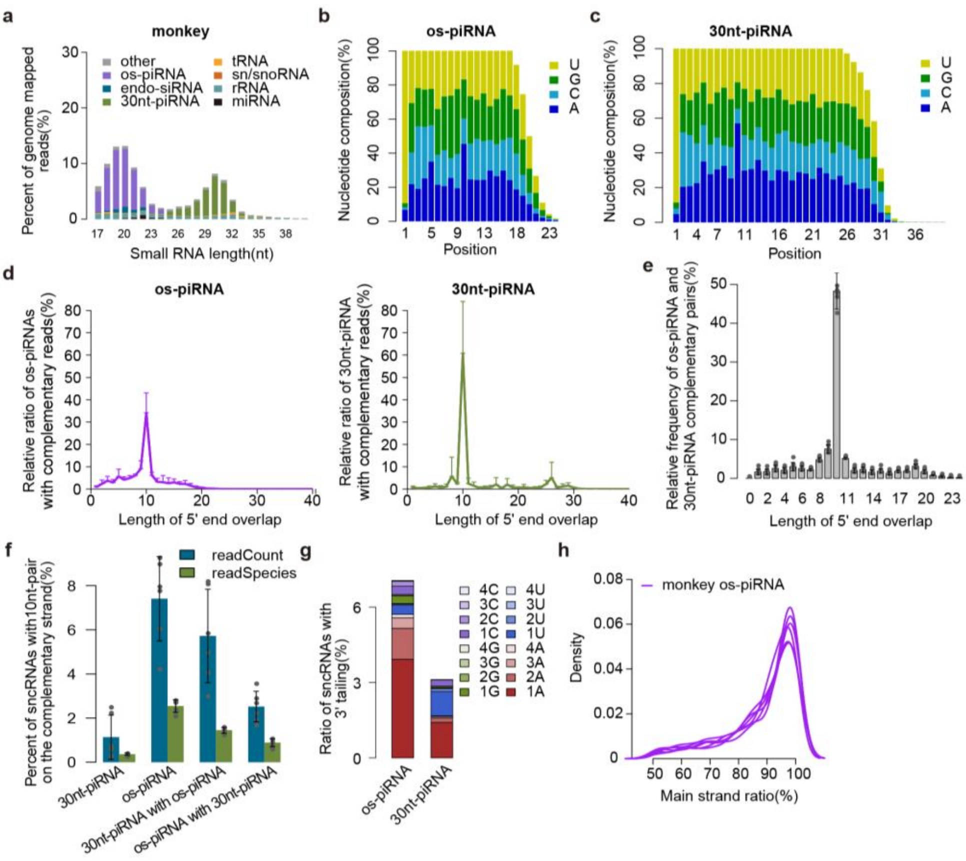

Supplementary Figure 8. Characteristics of small RNAs in monkey oocytes

(a) Composition of small RNA categories according to their length distributions in monkey oocytes. (b, c) The relative nucleotide composition of each position in monkey os-piRNAs (b) and 30-nt piRNAs (c). (d) Proportion of complementary small RNAs with different lengths of 5' overlaps in os-piRNAs and in 30-nt piRNAs of monkey oocytes. (e) The percentage of complementary os-piRNA and 30-nt piRNA pairs with different lengths of 5' end overlaps in monkey oocytes is shown. (f) The percentage of small RNAs harbouring the 10 nt 5’ overlap signature in monkey oocytes is shown. The results from the 30-nt piRNAs, the os-piRNAs, or the combination of these two kinds of small RNAs are shown. (g) The ratio of small RNAs tailed with mono- and oligo-nucleotides in monkey oocytes is shown. The average results for six monkey oocyte replicate samples are shown in (a-g), and the error bars in (d-f) represent the standard deviation. (h) The strand bias of monkey os-piRNAs in the clusters is shown. The results of six replicate samples are shown. Source data of (a-h) are provided in the Source Data file.

Supplementary References

1. Agarwal, V., Bell, G.W., Nam, J.W. & Bartel, D.P. Predicting effective microRNA target sites in mammalian mRNAs. *Elife* **4** (2015).
2. Sigrist, C.J.A. *et al.* PROSITE, a protein domain database for functional characterization and annotation. *Nucleic Acids Research* **38**, D161-D166 (2010).
3. Thompson, J.D., Gibson, T.J. & Higgins, D.G. Multiple sequence alignment using ClustalW and ClustalX. *Curr Protoc Bioinformatics* **Chapter 2**, Unit 2 3 (2002).
