# Supplementary material for: Single-cell CAS-seq reveals a class of short PIWI-interacting RNAs in human oocytes
Source: Nat Commun. 2019 Jul 29;10:3389. doi: 10.1038/s41467-019-11312-8 (PMC6662892; doi:10.1038/s41467-019-11312-8)
Supplement: Supplementary file 2 — Reporting summary [file 41467_2019_11312_MOESM2_ESM.pdf]

natureresearch

Corresponding author(s): Ligang Wu

Last updated by author(s): Jun 11, 2019

Reporting Summary

Nature Research wishes to improve the reproducibility of the work that we publish. This form provides structure for consistency and transparency in reporting. For further information on Nature Research policies, see Authors & Referees and the Editorial Policy Checklist.

Statistics

For all statistical analyses, confirm that the following items are present in the figure legend, table legend, main text, or Methods section.

n/a	Confirmed	
☐	☒	The exact sample size (n) for each experimental group/condition, given as a discrete number and unit of measurement
☐	☒	A statement on whether measurements were taken from distinct samples or whether the same sample was measured repeatedly
☐	☒	The statistical test(s) used AND whether they are one- or two-sided<br>*Only common tests should be described solely by name; describe more complex techniques in the Methods section.*
☐	☒	A description of all covariates tested
☐	☒	A description of any assumptions or corrections, such as tests of normality and adjustment for multiple comparisons
☐	☒	A full description of the statistical parameters including central tendency (e.g. means) or other basic estimates (e.g. regression coefficient) AND variation (e.g. standard deviation) or associated estimates of uncertainty (e.g. confidence intervals)
☐	☒	For null hypothesis testing, the test statistic (e.g. F, t, r) with confidence intervals, effect sizes, degrees of freedom and P value noted<br>*Give P values as exact values whenever suitable.*
☐	☒	For Bayesian analysis, information on the choice of priors and Markov chain Monte Carlo settings
☐	☒	For hierarchical and complex designs, identification of the appropriate level for tests and full reporting of outcomes
☐	☒	Estimates of effect sizes (e.g. Cohen's d, Pearson's r), indicating how they were calculated

Our web collection on statistics for biologists contains articles on many of the points above.

Software and code

Policy information about availability of computer code

Data collection	wget (version 1.14) and sratoolkit (version 2.5.4) was used to download and convert raw RNA sequencing data from SRA.
Data analysis	The softwares for small RNA mapping analyses include FASTX-Toolkit (version 0.0.13.1-1), bowtie (version 1.2.1.1), samtools (version 1.7), reallocate (version 1.0, http://www.smallrnagroup-mainz.de/software.html), Bedtools (version 2.21.0). proTRAC (version 2.1.2) was utilized to predict small RNA clusters. The conversion between different genome version or the identification of homologous regions was analyzed by liftOver. The softwares for RNA sequencing data analyses include trimmomatic (version 0.36) and hisat2 (2.0.5). The distribution of small RNA sequencing data on human genome was visualized with IGV (version 2.3.61). All of the statistical analyses were implemented with R (version 3.3.3). The prediction of os-piRNAs and endo-siRNAs and their clusters were implemented by custom algorithms which are available upon request.

For manuscripts utilizing custom algorithms or software that are central to the research but not yet described in published literature, software must be made available to editors/reviewers. We strongly encourage code deposition in a community repository (e.g. GitHub). See the Nature Research guidelines for submitting code & software for further information.

Data

Policy information about availability of data

All manuscripts must include a data availability statement. This statement should provide the following information, where applicable:

- Accession codes, unique identifiers, or web links for publicly available datasets
- A list of figures that have associated raw data
- A description of any restrictions on data availability

All sequencing data have been deposited in the Gene Expression Omnibus (GEO) of the National Center for Biotechnology Information (NCBI) under accession number GSE95218. The expression of miRNAs, os-piRNAs and piRNAs were provided in the supplementary data files. There is no restriction on data availability.

Field-specific reporting

Please select the one below that is the best fit for your research. If you are not sure, read the appropriate sections before making your selection.

☒ Life sciences ☐ Behavioural & social sciences ☐ Ecological, evolutionary & environmental sciences

For a reference copy of the document with all sections, see nature.com/documents/nr-reporting-summary-flat.pdf

Life sciences study design

All studies must disclose on these points even when the disclosure is negative.

Sample size

We profiled 91 samples in total, and the number of replicate samples for each experiment was listed in the supplementary table 1.
In addition, we downloaded the RNA sequencing data of two human oocyte samples detected by strand-specific library (GSE85632) and three human oocyte samples detected by strand-nonspecific library (GSE36552) from the NCBI Gene Expression Ominibus. To investigate the association of os-piRNA clusters with DNA methylation, the DNA methylation data of two human oocyte samples were downloaded from GSE49828.

The followings are short descriptions of sample size in our experiments.
To compare the sensitivity and reproducibility of CAS-seq with those of TruSeq (illumina) and 10ng-seq (our previous published method) in detecting miRNAs of HEK293 cell lines, two, three and three replicate samples were chosen for TruSeq, 10ng-seq and CAS-seq, respectively.
To compare the miRNA expressions detected by CAS-seq with those detected by qRT-PCR, two replicate samples of HEK293, A549 and Hela cell lines were chosen, respectively.
To compare the reproducibility of CAS-seq with that of 10ng-seq in detecting miRNAs of mouse oocyte, two, three and three replicate samples were chosen for 10ng-seq, 1ng CAS-seq (CAS-seq with 1ng total RNA) and single-oocyte CAS-seq (CAS-seq with single oocyte), respectively.
To detect the small non-coding RNAs in the oocyte of each species, six oocytes of human, crab-eating monkey and mouse were chosen separately.
To compare the os-piRNA expression detected by single oocyte and 1 ng of total RNA, 10 and 5 human GV oocytes were pooled to extract 1ng of total RNA, respectively. For mouse, 60 and 30 MII oocytes were pooled to extract 1ng of total RNA, respectively.
To investigate the influence of culture time on the expression of os-piRNAs in human GV oocyte , four replicate samples cultured by 2-4 hours and 23-25 hours were chosen, respectively. As the control of human MII oocyte, one sample of human cumulus cells was sequenced.
To identify the protein interacting with os-piRNAs in human oocyte, the IP input and output of 7 proteins (including rabbit IgG, Ago1-4, HIWI, and HIWI3) were profiled.
To investigate the modification of 2'-O-methylation at the 3' end of os-piRNA and 30nt-piRNA, three replicate samples of human oocyte before and after oxidization were profiled, respectively. As the control, two replicate samples of mouse oocyte before and after oxidization were also profiled, respectively.
To compare the $NaIO_4$ oxidization efficiency, four replicate samples of mouse testis before and after oxidization were profiled, respectively.
To investigate the dynamics of os-piRNA and 30nt-piRNA expression during human early development, three replicate samples of human oocyte, 2cell and morula embryo were profiled, respectively.

Data exclusions

No data were excluded from the analyses.

Replication

All attempts at replication were successful.

Randomization

No samples were randomly allocated into experimental groups in our study. For instance, the HEK293 samples were grouped based on the input amount of total RNA or the method to construct small RNA librarys. The MII oocyte samples of the same species were allocated into one group. The expression levels of small RNAs in each sample were normalized by RPM, and were utilized to evaluate the reproducibility of CAS-seq.

Blinding

All the samples of our study were collected cells or embryos. The small RNAs of each sample were profiled and analyzed independently.

Reporting for specific materials, systems and methods

We require information from authors about some types of materials, experimental systems and methods used in many studies. Here, indicate whether each material, system or method listed is relevant to your study. If you are not sure if a list item applies to your research, read the appropriate section before selecting a response.

Materials & experimental systems

n/a	Involved in the study
☐	☒ Antibodies
☐	☒ Eukaryotic cell lines
☒	☐ Palaeontology
☐	☒ Animals and other organisms
☐	☒ Human research participants
☒	☐ Clinical data

Methods

n/a	Involved in the study
☒	☐ ChIP-seq
☒	☐ Flow cytometry
☒	☐ MRI-based neuroimaging

Antibodies

Antibodies used	Antibodies used in immunoprecipitation were polyclonal antibodies from rabbit. The peptides, C-HDLGVNTRQNLDHVKESK, C-GKPEEPSTQRGPAQ and C-TRQDMKHVKDSKTGSEG were chosen as antigens to produce antibodies for HIWI, HILI and HIWI3 respectively.
Validation	The antibodies were validated to specifically interact with the target proteins. The Antibody immunoprecipitation assay was described in the method part.

Eukaryotic cell lines

Policy information about cell lines

Cell line source(s)	HEK293, Hela, and A549 cells were purchased from ATCC (Maryland, USA).
Authentication	The cell lines used have been authenticated by the sourced company.
Mycoplasma contamination	All the cell lines were tested to be negative for mycoplasma contamination.
Commonly misidentified lines (See ICLAC register)	No commonly misidentified cell lines were used.

Animals and other organisms

Policy information about studies involving animals; ARRIVE guidelines recommended for reporting animal research

Laboratory animals	mice for oocyte collection: B6D2F1(female C57BL/6 x male DBA/2) 8-10 week<br>mice for testis collection: male C57BL/6J, 18.5 day<br>monkeys: Adult female Macaca fascicularis monkeys
Wild animals	No wild animals were involved.
Field-collected samples	No samples collected from the field were involved.
Ethics oversight	Animal procedures were conducted in compliance with the ethical guidelines of the Shanghai Institute of Biochemistry and Cell Biology and received ethical approval from the Shanghai Institute of Biochemistry and Cell Biology for acquiring mouse oocytes and mouse testis. Monkey oocytes collection was conducted in compliance with the ethical guidelines of the Institute of Neuroscience and received ethical approval from the Institute of Neuroscience. Shanghai Institutes for Biological Sciences, Chinese Academy of Sciences.

Note that full information on the approval of the study protocol must also be provided in the manuscript.

Human research participants

Policy information about studies involving human research participants

Population characteristics	Female adult patients of Asian<br>Male adult patients of Asian
Recruitment	Patients were recruited with no bias from volunteers wishing to participate in research studies.
Ethics oversight	The study of human oocyte and embryo has been approved by the Reproductive Study Ethics Committee of Shanghai Ninth Hospital (Research license 20161206). The informed consent process for the embryos and gametes donated complied with the clinical protocols of Department of Assisted Reproduction, Shanghai Ninth People's Hospital Affiliated to Shanghai Jiao Tong University School of Medicine.<br>The study of human testis has been approved by the Reproductive Study Ethics Committee of China National Population and Family Planning Key Laboratory of Contraceptive Drugs and Devices, Shanghai Institute of Planned Parenthood Research (SIPPR).

The informed consent process for the testis donated complied with the clinical protocols of China National Population and Family Planning Key Laboratory of Contraceptive Drugs and Devices, Shanghai Institute of Planned Parenthood Research (SIPPR).

Note that full information on the approval of the study protocol must also be provided in the manuscript.
