# Supplementary material for: Single-cell CAS-seq reveals a class of short PIWI-interacting RNAs in human oocytes
Source: Nat Commun. 2019 Jul 29;10:3389. doi: 10.1038/s41467-019-11312-8 (PMC6662892; doi:10.1038/s41467-019-11312-8)
Supplement: Supplementary file 3 — Description of Additional Supplementary Files [file 41467_2019_11312_MOESM3_ESM.docx]

**Description of Additional Supplementary Files**

File Name: Supplementary Data 1.
Description: Expression of miRNAs in human HEK293 and other cell lines

File Name: Supplementary Data 2.
Description: Summary of small RNA sequencing data
File Name: Supplementary Data 3.
Description: Expression of miRNAs in mouse oocyte

File Name: Supplementary Data 4.
Description: Expression of 30-nt piRNA sequences in human oocyte and early embryos

File Name: Supplementary Data 5.
Description: Expression of 30-nt piRNA clusters in human oocyte and early embryos

File Name: Supplementary Data 6.
Description: Expression of os-piRNA sequences in human oocyte and early embryos

File Name: Supplementary Data 7.
Description: Expression of os-piRNA clusters in human oocyte and early embryos

File Name: Supplementary Data 8.
Description: Expression of os-piRNA clusters in monkey oocyte

File Name: Supplementary Data 9.
Description: Homologous relationship between human and monkey os-piRNA clusters

File Name: Supplementary Data 10.
Description: The expression of os-piRNAs and 30nt-piRNAs derived from TEs in human oocyte and early embryos

File Name: Supplementary Data 11.
Description: The summary of primer and oligo sequences
